# Supplementary material for: Selection of Polymorphic Patterns Obtained by RAPD-PCR through Qualitative and Quantitative Analyses to Differentiate Aspergillus fumigatus
Source: J Fungi (Basel). 2022 Mar 13;8(3):296. doi: 10.3390/jof8030296 (PMC8949193; doi:10.3390/jof8030296)
Supplement: Supplementary file 1 [file jof-08-00296-s001.zip › Supplementary Table S1.pdf]

Journal of Fungi

MDPI

Tables S1 (1-3). Mathematical assessment and testing to predict Aspergillus species

Table 1. Evaluation of the probability equation with primer OPC-06 of Cuban isolates to determine the most probable species with the statistical analysis.

OPC-06

AISLADO	PRIMER	BANDAS	pb	Intensidad	Correspondencia *Flavus*	Correspondencia *Fumigatus*	Correspondencia *Niger*	Correspondencia *Tubingesis*	Especie más probable
PRU1FUMCUBANO	OPC-06	1	150	1	**0.87562731**	0.00934631	0.11199239	0.00303399	FLAVUS
PRU1FUMCUBANO	OPC-06	2	200	0.5	**0.8766509**	0.00732215	0.11346158	0.00256537	FLAVUS
PRU1FUMCUBANO	OPC-06	3	300	0.5	**0.86484428**	0.00926619	0.12299141	0.00289813	FLAVUS
PRU1FUMCUBANO	OPC-06	4	400	0.5	**0.85190284**	0.01170859	0.13311948	0.00326908	FLAVUS
PRU1FUMCUBANO	OPC-06	5	500	0.5	**0.83771509**	0.01476938	0.14383435	0.00368119	FLAVUS
PRU1FUMCUBANO	OPC-06	6	550	0.5	**0.83011632**	0.01657602	0.14940412	0.00390354	FLAVUS
PRU1FUMCUBANO	OPC-06	7	700	0.5	**0.80510627**	0.02335724	0.16689714	0.00463935	FLAVUS
PRU1FUMCUBANO	OPC-06	8	1000	0.5	**0.74355205**	0.04553377	0.20448015	0.00643404	FLAVUS
PRU1FUMCUBANO	OPC-06	9	1200	0.5	**0.69245395**	0.06977754	0.22991118	0.00785733	FLAVUS
PRU1FUMCUBANO	OPC-06	1	150	1	**0.87562731**	0.00934631	0.11199239	0.00303399	FLAVUS
PRU1FUMCUBANO	OPC-06	2	200	0.5	**0.8766509**	0.00732215	0.11346158	0.00256537	FLAVUS
PRU1FUMCUBANO	OPC-06	3	300	0.5	**0.86484428**	0.00926619	0.12299141	0.00289813	FLAVUS
PRU1FUMCUBANO	OPC-06	4	400	0.5	**0.85190284**	0.01170859	0.13311948	0.00326908	FLAVUS
PRU1FUMCUBANO	OPC-06	5	500	0.5	**0.83771509**	0.01476938	0.14383435	0.00368119	FLAVUS
PRU1FUMCUBANO	OPC-06	6	550	0.5	**0.83011632**	0.01657602	0.14940412	0.00390354	FLAVUS
PRU1FUMCUBANO	OPC-06	7	700	0.5	**0.80510627**	0.02335724	0.16689714	0.00463935	FLAVUS
PRU1FUMCUBANO	OPC-06	8	1000	1	**0.72229925**	0.06402121	0.20576021	0.00791934	FLAVUS
PRU1FUMCUBANO	OPC-06	9	1200	1	**0.66482232**	0.09696497	0.22865422	0.00955849	FLAVUS
PRU1FUMCUBANO	OPC-06	1	150	1	**0.87562731**	0.00934631	0.11199239	0.00303399	FLAVUS
PRU1FUMCUBANO	OPC-06	2	200	0.5	**0.8766509**	0.00732215	0.11346158	0.00256537	FLAVUS
PRU1FUMCUBANO	OPC-06	3	300	0.5	**0.86484428**	0.00926619	0.12299141	0.00289813	FLAVUS

PRU1FUMCUBANO	OPC-06	4	400	0.5	**0.85190284**	0.01170859	0.13311948	0.00326908	FLAVUS
PRU1FUMCUBANO	OPC-06	5	500	0.5	**0.83771509**	0.01476938	0.14383435	0.00368119	FLAVUS
PRU1FUMCUBANO	OPC-06	6	550	0.5	**0.83011632**	0.01657602	0.14940412	0.00390354	FLAVUS
PRU1FUMCUBANO	OPC-06	7	700	0.5	**0.80510627**	0.02335724	0.16689714	0.00463935	FLAVUS
PRU1FUMCUBANO	OPC-06	8	1000	2	**0.66270937**	0.12305487	0.2025705	0.01166525	FLAVUS
PRU1FUMCUBANO	OPC-06	9	1200	1	**0.66482232**	0.09696497	0.22865422	0.00955849	FLAVUS
PRU1FUMCUBANO	OPC-06	1	150	0.5	**0.88216084**	0.00650555	0.10892124	0.00241237	FLAVUS
PRU1FUMCUBANO	OPC-06	2	200	0.5	**0.8766509**	0.00732215	0.11346158	0.00256537	FLAVUS
PRU1FUMCUBANO	OPC-06	3	300	0.5	**0.86484428**	0.00926619	0.12299141	0.00289813	FLAVUS
PRU1FUMCUBANO	OPC-06	4	400	0.5	**0.85190284**	0.01170859	0.13311948	0.00326908	FLAVUS
PRU1FUMCUBANO	OPC-06	5	500	0.5	**0.83771509**	0.01476938	0.14383435	0.00368119	FLAVUS
PRU1FUMCUBANO	OPC-06	6	550	0.5	**0.83011632**	0.01657602	0.14940412	0.00390354	FLAVUS
PRU1FUMCUBANO	OPC-06	7	700	0.5	**0.80510627**	0.02335724	0.16689714	0.00463935	FLAVUS
PRU1FUMCUBANO	OPC-06	8	1000	1	**0.72229925**	0.06402121	0.20576021	0.00791934	FLAVUS
PRU1FUMCUBANO	OPC-06	9	1200	1	**0.66482232**	0.09696497	0.22865422	0.00955849	FLAVUS

Table 2. Evaluation of the probability equation with primer OPF-O1 of Cuban isolates to determine the most probable species with the statistical analysis.

OPF-01

AISLADO	PRIMER	CODIGO	BANDAS	pb	Intensidad	Correspondencia *Flavus*	Correspondencia *Fumigatus*	Correspondencia *Niger*	Correspondencia *Tubingesis*	Especie más probable
PRU3FUMCUBANO	OPF-01	FC1	1	450	0.5	2.07E-10	**1**	3.57E-11	1.44E-10	FUMIGATUS
PRU3FUMCUBANO	OPF-01	FC1	2	500	0.5	1.99E-10	**1**	3.61E-11	1.42E-10	FUMIGATUS
PRU3FUMCUBANO	OPF-01	FC1	3	600	1	2.47E-11	**1**	5.94E-12	2.35E-11	FUMIGATUS
PRU3FUMCUBANO	OPF-01	FC1	4	800	0.5	1.58E-10	**1**	3.81E-11	1.26E-10	FUMIGATUS
PRU3FUMCUBANO	OPF-01	FC1	5	900	2	3.54E-13	**1**	1.64E-13	6.26E-13	FUMIGATUS
PRU3FUMCUBANO	OPF-01	FC1	6	1000	2	3.28E-13	**1**	1.67E-13	6.01E-13	FUMIGATUS
PRU3FUMCUBANO	OPF-01	FC1	7	1000	2	3.28E-13	**1**	1.67E-13	6.01E-13	FUMIGATUS

Journal of Fungi

MDPI

PRU3FUMCUBANO	OPF-01	FC1	8	1200	0.5	1.16E-10	**1**	4.10E-11	1.07E-10	FUMIGATUS
PRU3FUMCUBANO	OPF-01	FC1	9	1300	1	1.45E-11	**1**	6.75E-12	1.78E-11	FUMIGATUS
PRU3FUMCUBANO	OPF-01	FC2	1	450	0.5	2.07E-10	**1**	3.57E-11	1.44E-10	FUMIGATUS
PRU3FUMCUBANO	OPF-01	FC2	2	500	0.5	1.99E-10	**1**	3.61E-11	1.42E-10	FUMIGATUS
PRU3FUMCUBANO	OPF-01	FC2	3	600	1	2.47E-11	**1**	5.94E-12	2.35E-11	FUMIGATUS
PRU3FUMCUBANO	OPF-01	FC2	4	800	0.5	1.58E-10	**1**	3.81E-11	1.26E-10	FUMIGATUS
PRU3FUMCUBANO	OPF-01	FC2	5	900	2	3.54E-13	**1**	1.64E-13	6.26E-13	FUMIGATUS
PRU3FUMCUBANO	OPF-01	FC2	6	1000	2	3.28E-13	**1**	1.67E-13	6.01E-13	FUMIGATUS
PRU3FUMCUBANO	OPF-01	FC2	7	1000	2	3.28E-13	**1**	1.67E-13	6.01E-13	FUMIGATUS
PRU3FUMCUBANO	OPF-01	FC2	8	1200	0.5	1.16E-10	**1**	4.10E-11	1.07E-10	FUMIGATUS
PRU3FUMCUBANO	OPF-01	FC2	9	1300	1	1.45E-11	**1**	6.75E-12	1.78E-11	FUMIGATUS
PRU3FUMCUBANO	OPF-01	FC3	1	450	0.5	2.07E-10	**1**	3.57E-11	1.44E-10	FUMIGATUS
PRU3FUMCUBANO	OPF-01	FC3	2	500	0.5	1.99E-10	**1**	3.61E-11	1.42E-10	FUMIGATUS
PRU3FUMCUBANO	OPF-01	FC3	3	600	1	2.47E-11	**1**	5.94E-12	2.35E-11	FUMIGATUS
PRU3FUMCUBANO	OPF-01	FC3	4	800	0.5	1.58E-10	**1**	3.81E-11	1.26E-10	FUMIGATUS
PRU3FUMCUBANO	OPF-01	FC3	5	900	2	3.54E-13	**1**	1.64E-13	6.26E-13	FUMIGATUS
PRU3FUMCUBANO	OPF-01	FC3	6	1000	2	3.28E-13	**1**	1.67E-13	6.01E-13	FUMIGATUS
PRU3FUMCUBANO	OPF-01	FC3	7	1000	2	3.28E-13	**1**	1.67E-13	6.01E-13	FUMIGATUS
PRU3FUMCUBANO	OPF-01	FC3	8	1200	0.5	1.16E-10	**1**	4.10E-11	1.07E-10	FUMIGATUS
PRU3FUMCUBANO	OPF-01	FC3	9	1300	1	1.45E-11	**1**	6.75E-12	1.78E-11	FUMIGATUS
PRU3FUMCUBANO	OPF-01	FC4	1	450	0.5	2.07E-10	**1**	3.57E-11	1.44E-10	FUMIGATUS
PRU3FUMCUBANO	OPF-01	FC4	2	500	0.5	1.99E-10	**1**	3.61E-11	1.42E-10	FUMIGATUS
PRU3FUMCUBANO	OPF-01	FC4	3	600	1	2.47E-11	**1**	5.94E-12	2.35E-11	FUMIGATUS
PRU3FUMCUBANO	OPF-01	FC4	4	800	0.5	1.58E-10	**1**	3.81E-11	1.26E-10	FUMIGATUS
PRU3FUMCUBANO	OPF-01	FC4	5	900	2	3.54E-13	**1**	1.64E-13	6.26E-13	FUMIGATUS
PRU3FUMCUBANO	OPF-01	FC4	6	1000	2	3.28E-13	**1**	1.67E-13	6.01E-13	FUMIGATUS
PRU3FUMCUBANO	OPF-01	FC4	7	1000	2	3.28E-13	**1**	1.67E-13	6.01E-13	FUMIGATUS
PRU3FUMCUBANO	OPF-01	FC4	8	1200	0.5	1.16E-10	**1**	4.10E-11	1.07E-10	FUMIGATUS
PRU3FUMCUBANO	OPF-01	FC4	9	1300	1	1.45E-11	**1**	6.75E-12	1.78E-11	FUMIGATUS

Journal of Fungi

MDPI

Table 3. Evaluation of the probability equation with primer OPG-13 of Cuban isolates to determine the most probable species with the statistical analysis.

OPG-13

AISLADO	PRIMER	CODIGO	BANDAS	pb	Intensidad	Correspondencia *Flavus*	Correspondencia *Fumigatus*	Correspondencia *Niger*	Correspondencia *Tubingensis*	Especie más probable
PRU1FUMCUBANO	OPG-13	TubC1	1	450	0.5	0.03623853	0.00314777	0.00060975	**0.96000395**	TUBINGENSIS
PRU1FUMCUBANO	OPG-13	TubC1	2	500	0.5	0.03060085	0.0025014	0.00052325	**0.96637449**	TUBINGENSIS
PRU1FUMCUBANO	OPG-13	TubC2	1	450	0.5	0.03623853	0.00314777	0.00060975	**0.96000395**	TUBINGENSIS
PRU1FUMCUBANO	OPG-13	TubC2	2	500	0.5	0.03060085	0.0025014	0.00052325	**0.96637449**	TUBINGENSIS
PRU1FUMCUBANO	OPG-13	TubC3	1	450	0.5	0.03623853	0.00314777	0.00060975	**0.96000395**	TUBINGENSIS
PRU1FUMCUBANO	OPG-13	TubC3	2	500	0.5	0.03060085	0.0025014	0.00052325	**0.96637449**	TUBINGENSIS
PRU1FUMCUBANO	OPG-13	NC1	1	1200	0.5	0.00046182	9.67E-06	4.04E-06	**0.99952447**	TUBINGENSIS
PRU1FUMCUBANO	OPG-13	NC2	1	1200	0.5	0.00046182	9.67E-06	4.04E-06	**0.99952447**	TUBINGENSIS
