# Supplementary material for: Selection of Polymorphic Patterns Obtained by RAPD-PCR through Qualitative and Quantitative Analyses to Differentiate Aspergillus fumigatus
Source: J Fungi (Basel). 2022 Mar 13;8(3):296. doi: 10.3390/jof8030296 (PMC8949193; doi:10.3390/jof8030296)
Supplement: Supplementary file 1 [file jof-08-00296-s001.zip › Supplementary Figure S1.pdf]

Journal of Fungi

MDPI

Figures S1 . Macro and micro morphological characteristics of the isolates used in this work.

	MACROSCOPIC MORPHOLOGY		MICROSCOPIC MORPHOLOGY
SECTION	OBVERSE	BACK	
Flavi			
323C	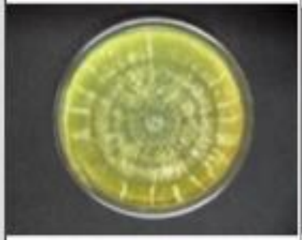	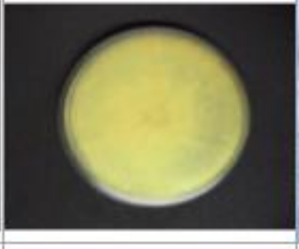	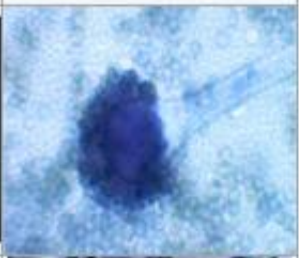
87A	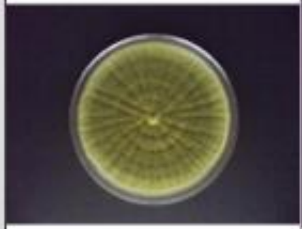	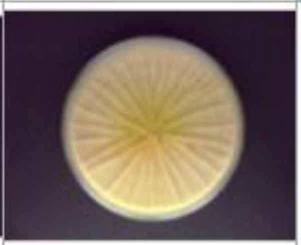	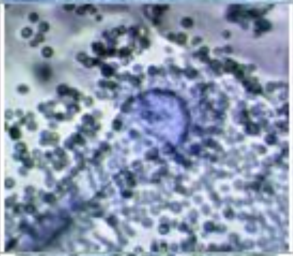
A40	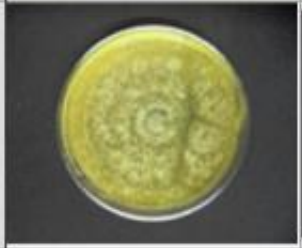	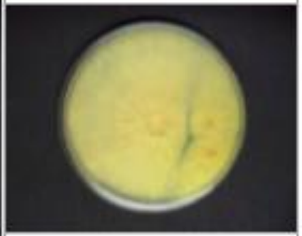	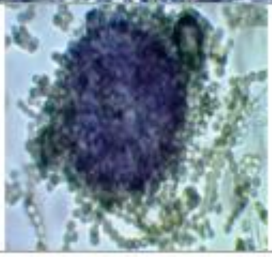
370B	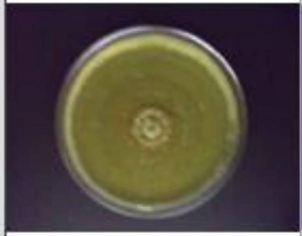	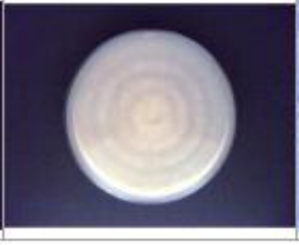	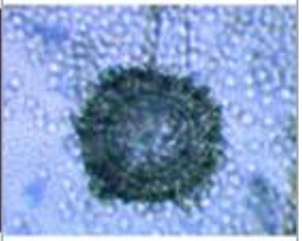
Fumigati			
MM12	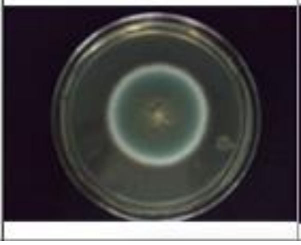	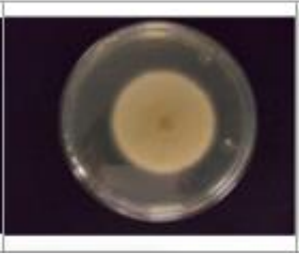	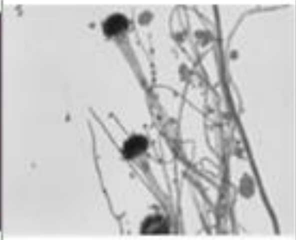

Journal of *Fungi*

MDPI

	MACROSCOPIC MORPHOLOGY		MICROSCOPIC MORPHOLOGY
MM16	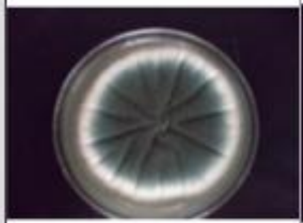	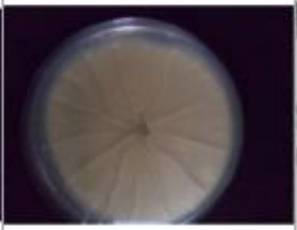	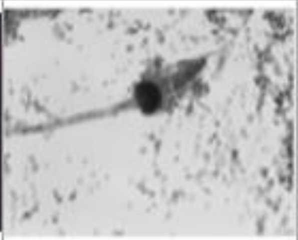
MM18	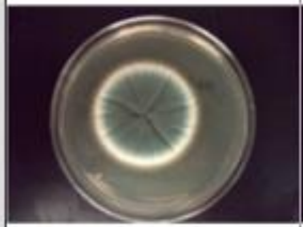	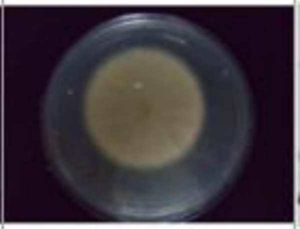	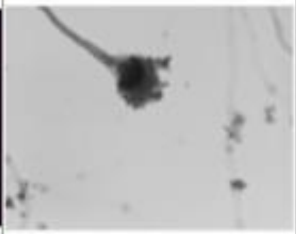
MM21	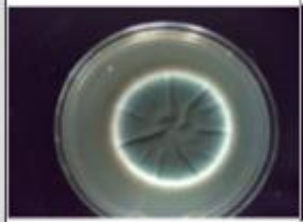	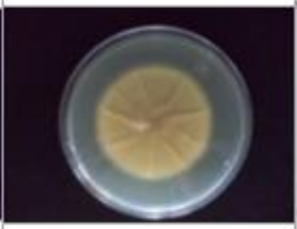	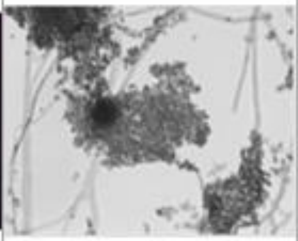
Nigri			
387A	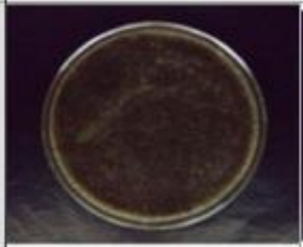	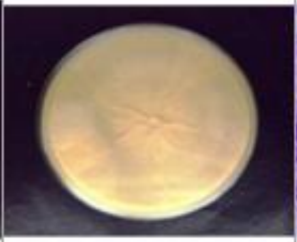	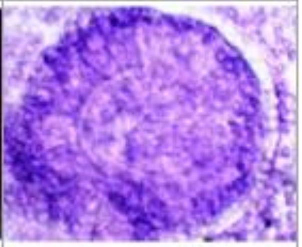
A39	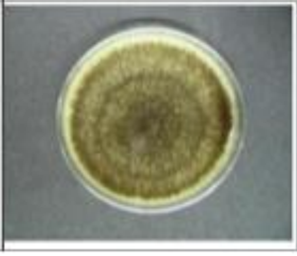	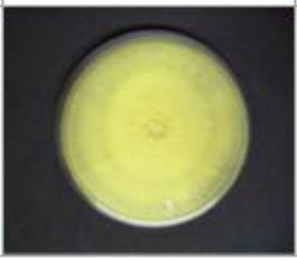	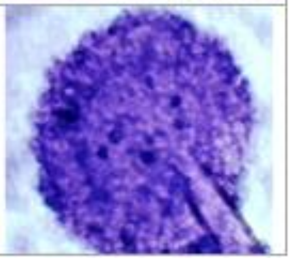

Journal of
Fungi

MDPI

	MACROSCOPIC MORPHOLOGY		MICROSCOPIC MORPHOLOGY
335B	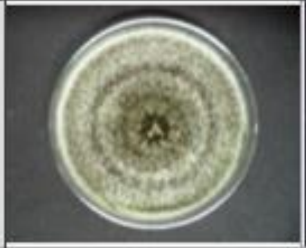	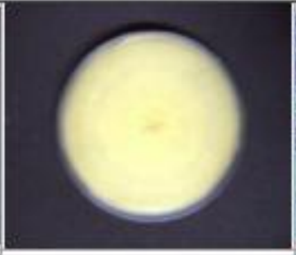	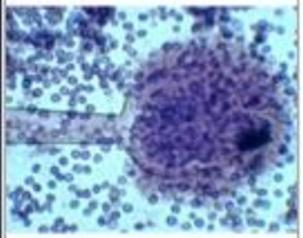
88C	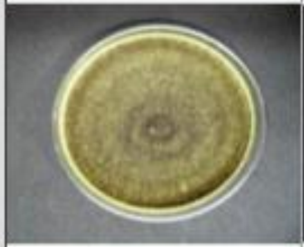	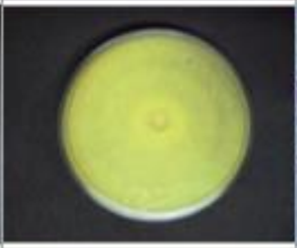	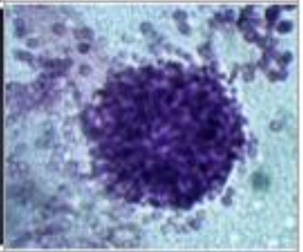
281C	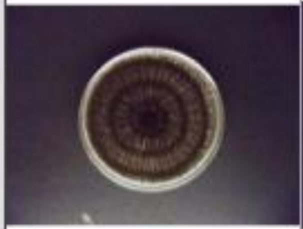	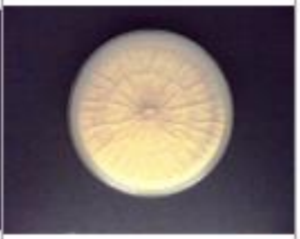	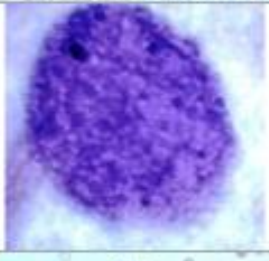
146A	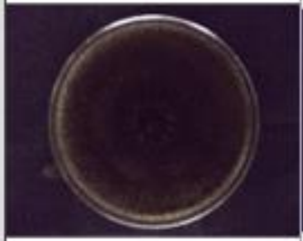	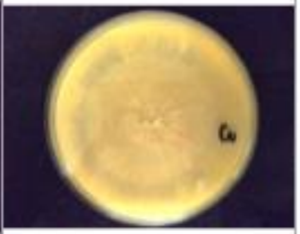	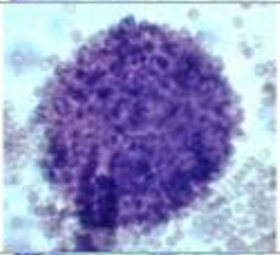
A26	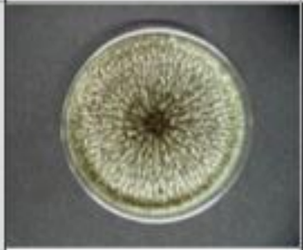	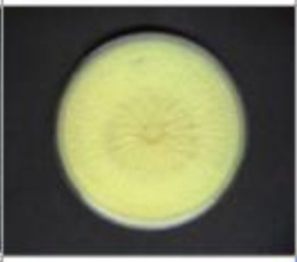	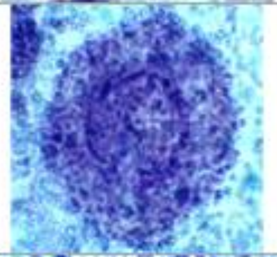
220A	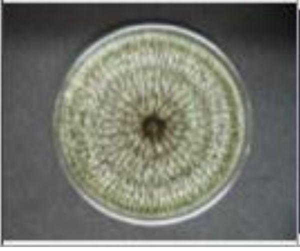	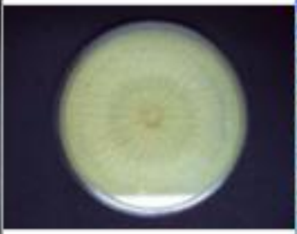	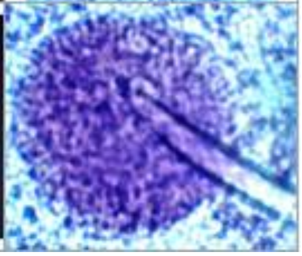
