# Supplementary material for: Selection of Polymorphic Patterns Obtained by RAPD-PCR through Qualitative and Quantitative Analyses to Differentiate Aspergillus fumigatus
Source: J Fungi (Basel). 2022 Mar 13;8(3):296. doi: 10.3390/jof8030296 (PMC8949193; doi:10.3390/jof8030296)
Supplement: Supplementary file 1 [file jof-08-00296-s001.zip › Supplementary Figure S2.pdf]

Journal of Fungi

MDPI

**Figures S2 (1-43). Polymorphic patterns obtained by RAPD-PCR for the four species with the 34 oligonucleotides**

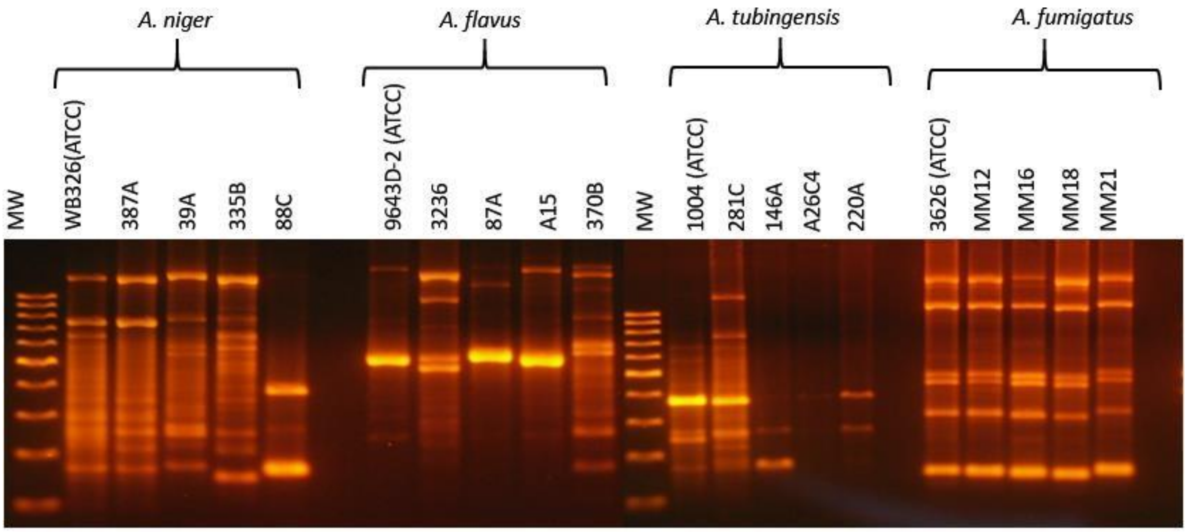


**Figure 1.** RAPD-PCR of polymorphic patterns of *Aspergillus* isolates with primer OPA-19.

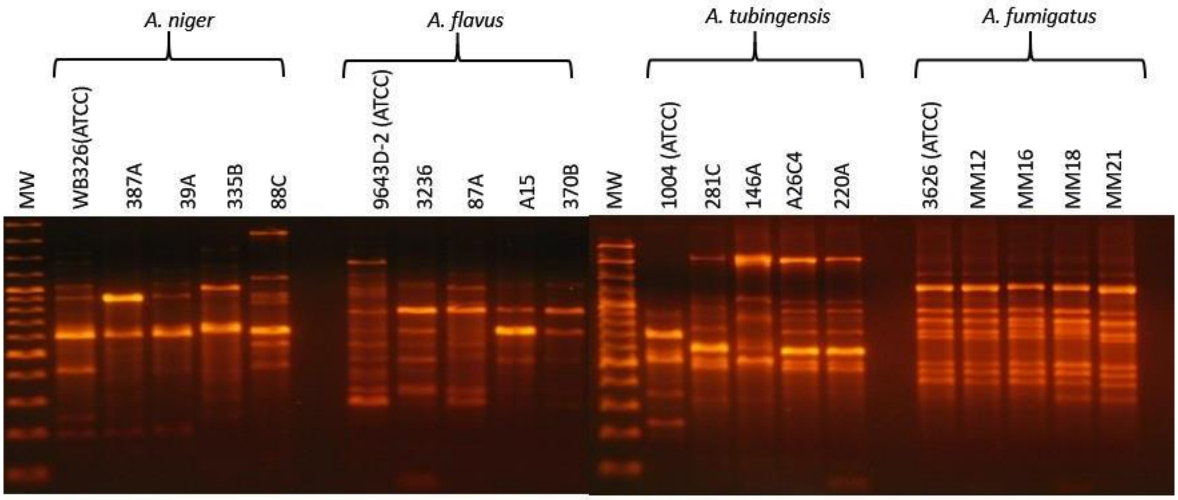


**Figure 2.** RAPD-PCR of polymorphic patterns of *Aspergillus* isolates with primer OPF-01.

Journal of Fungi

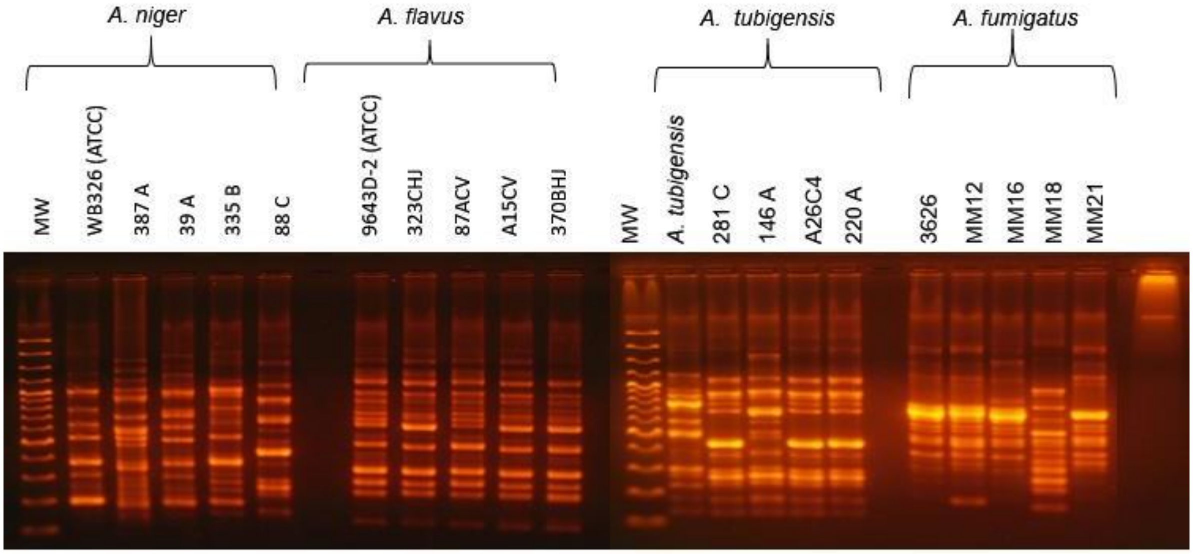


**Figure 3.** RAPD-PCR of polymorphic patterns of *Aspergillus* isolates with primer P54.

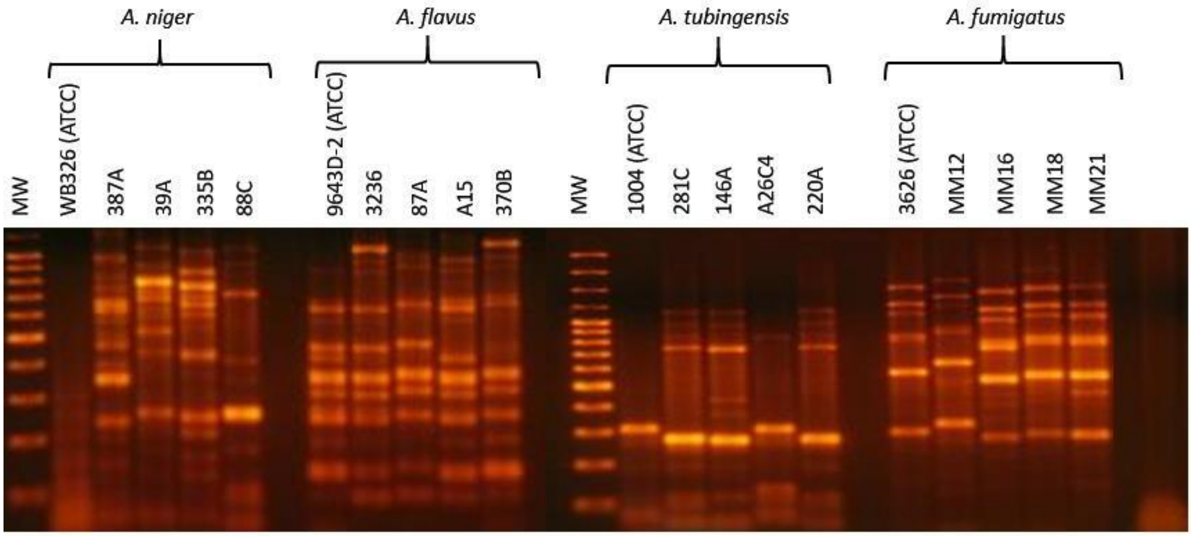


**Figure 4.** RAPD-PCR of polymorphic patterns of *Aspergillus* isolates with primer OPC-06.

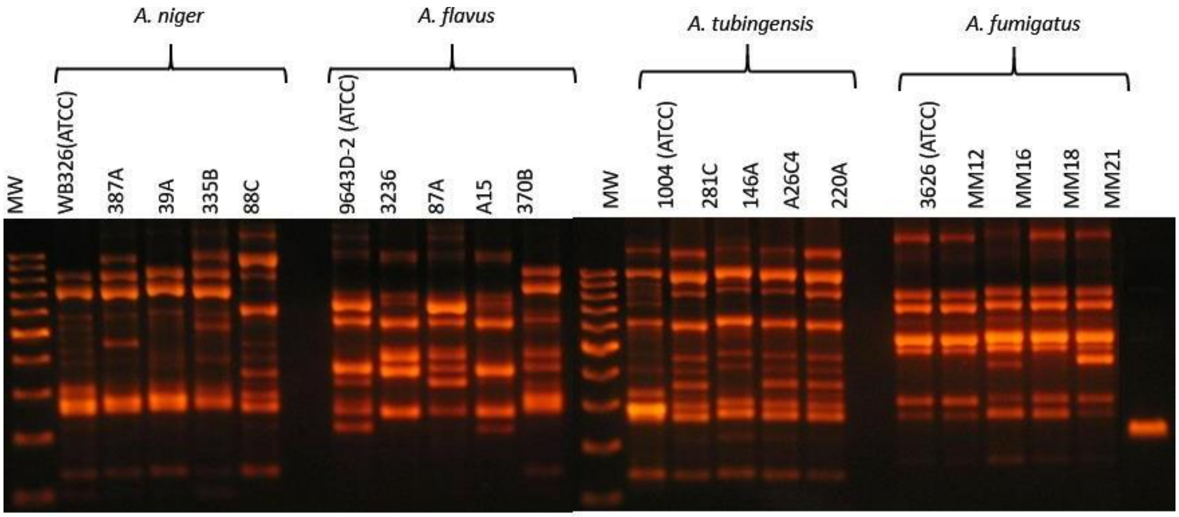


**Figure 5.** RAPD-PCR of polymorphic patterns of *Aspergillus* isolates with primer 1253.

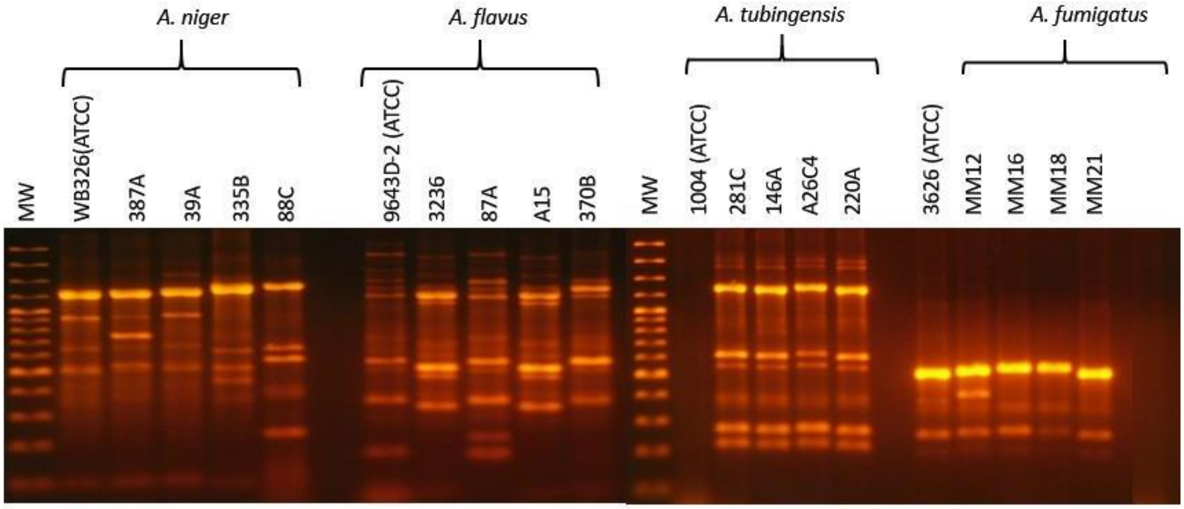


**Figure 6.** RAPD-PCR of polymorphic patterns of *Aspergillus* isolates with primer OPF-07.

Journal of Fungi

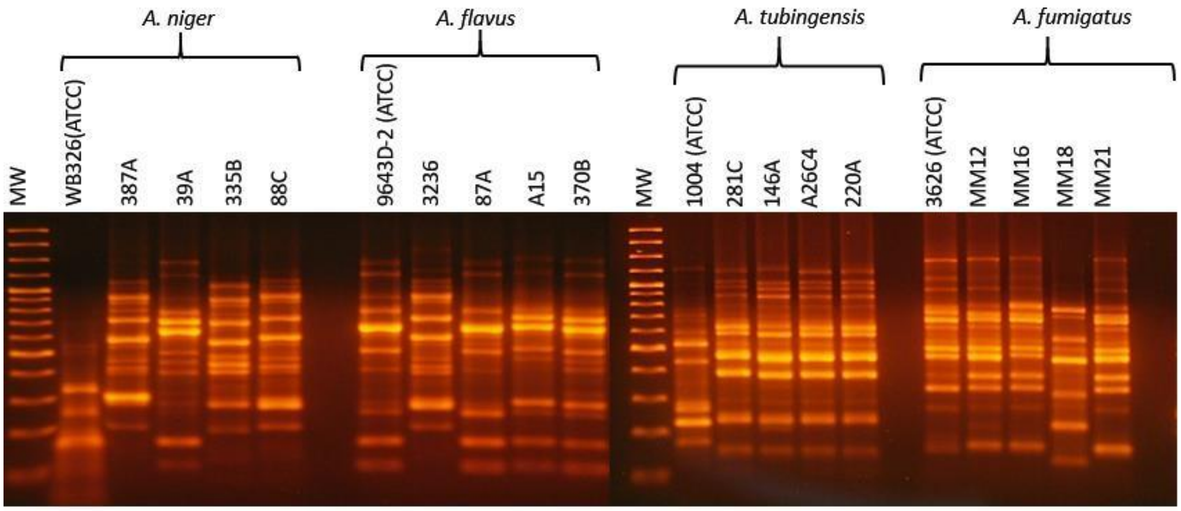


**Figure 7.** RAPD-PCR of polymorphic patterns of *Aspergillus* isolates with primer OPA-02.

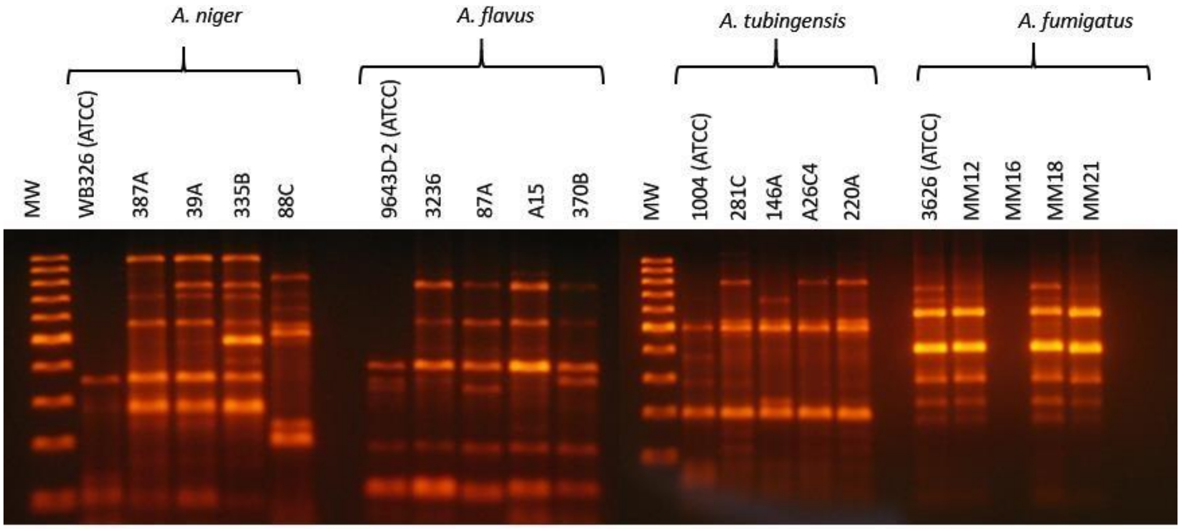


**Figure 8**. RAPD-PCR of polymorphic patterns of *Aspergillus* isolates with primer OPG-13.

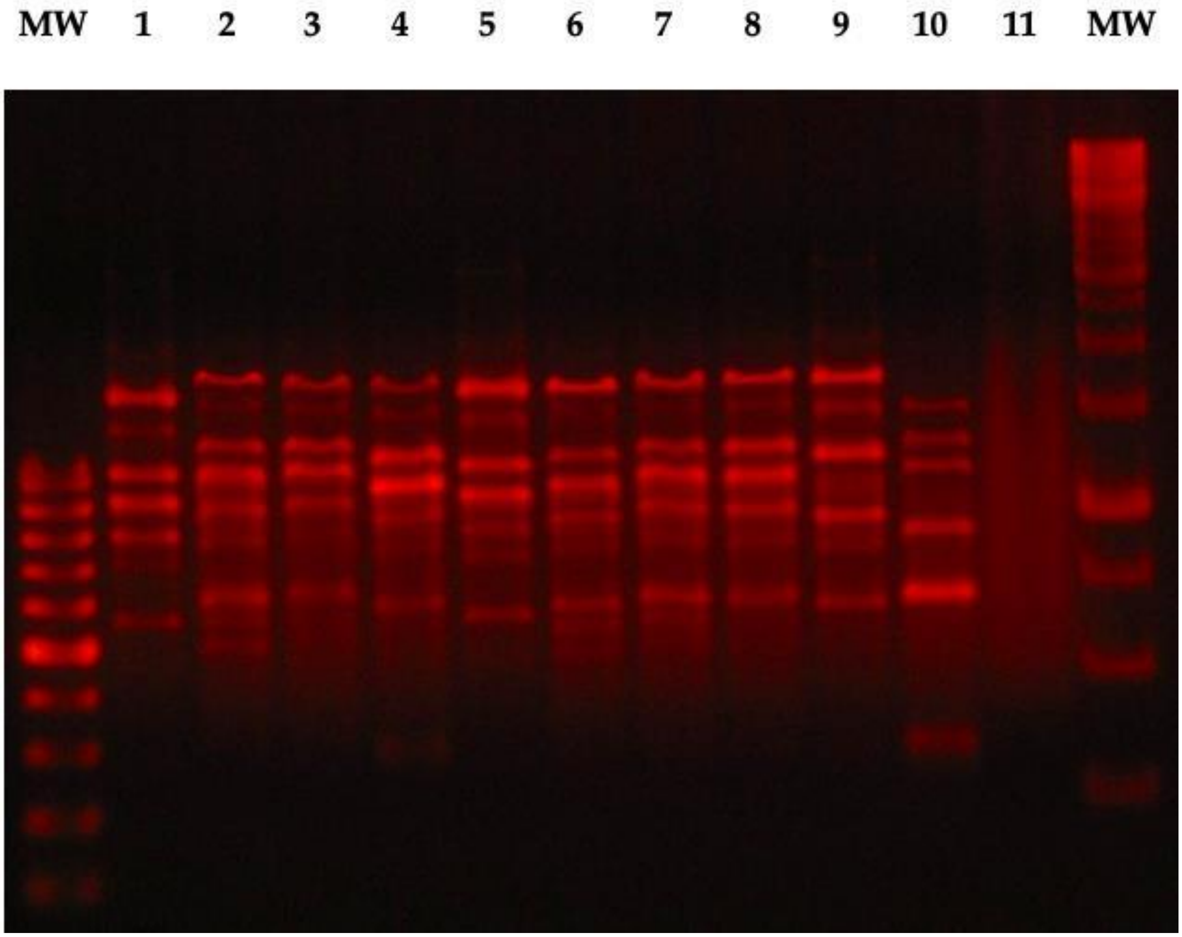


**Figure 9**. Comparison of polymorphic patterns of *A. fumigatus* isolates of different geographic origin and *A. lentulus* with primer OPF-01. Reference strain (ATCC) (line 1), Mexican isolates (line 2-5), Cuban isolates (6-9), *A. lentulus* (ATCC) (line 10).

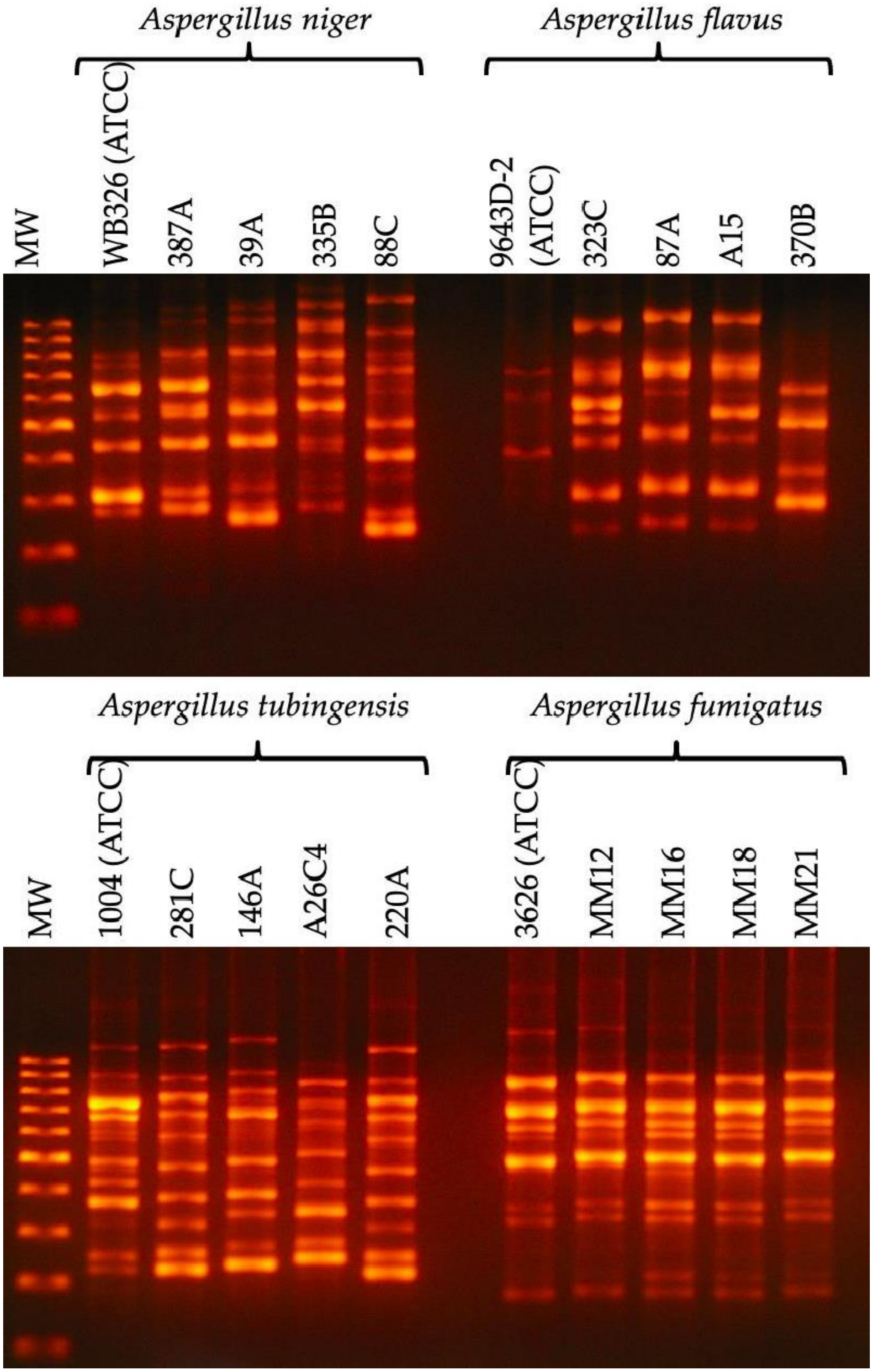


**Figure 10**. Polymorphic patterns obtained by RAPD-PCR for the four species with oligonucleotide 1281.

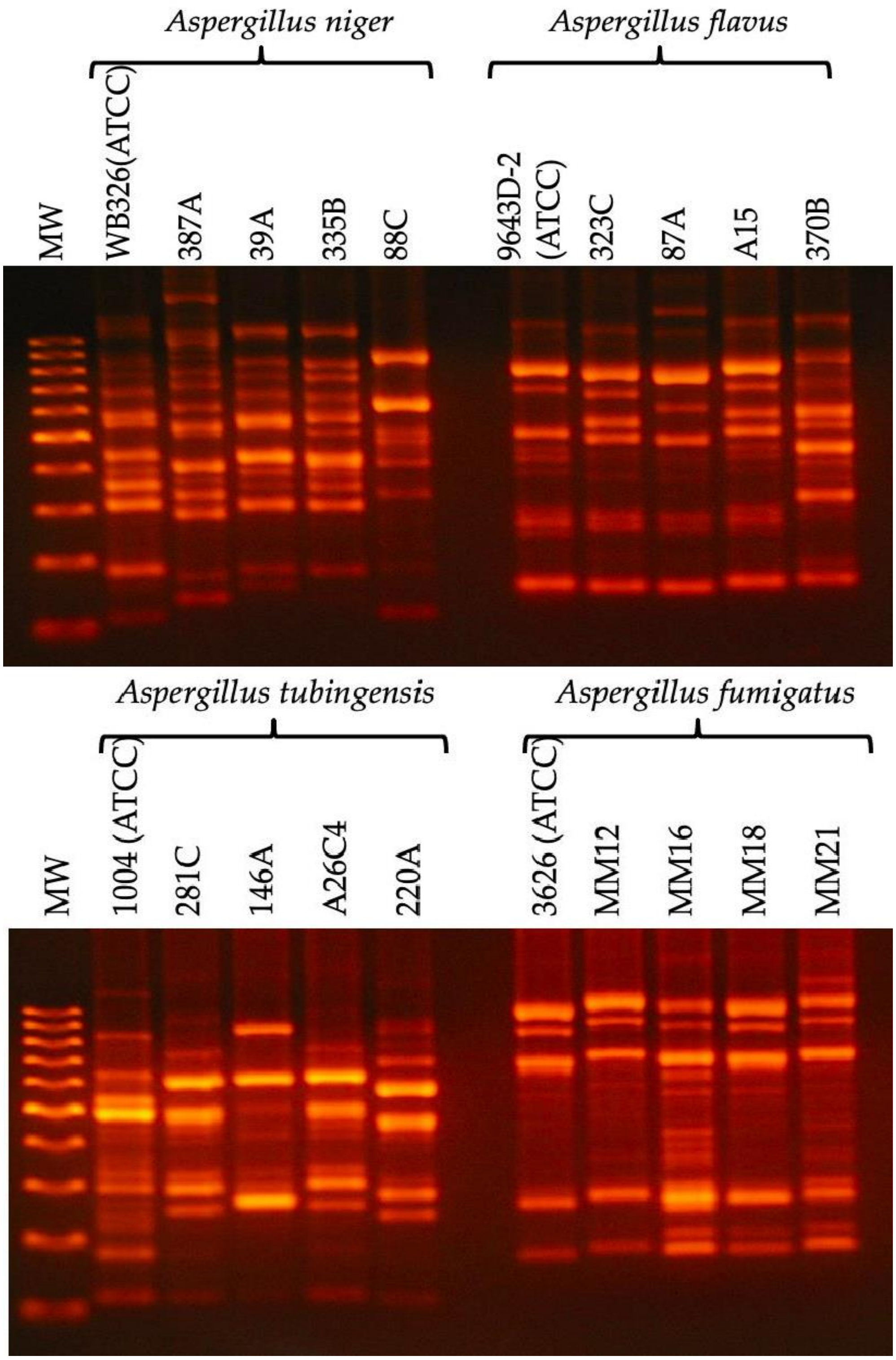


**Figure 11**. Polymorphic patterns obtained by RAPD-PCR for the four species with oligonucleotide 1283.

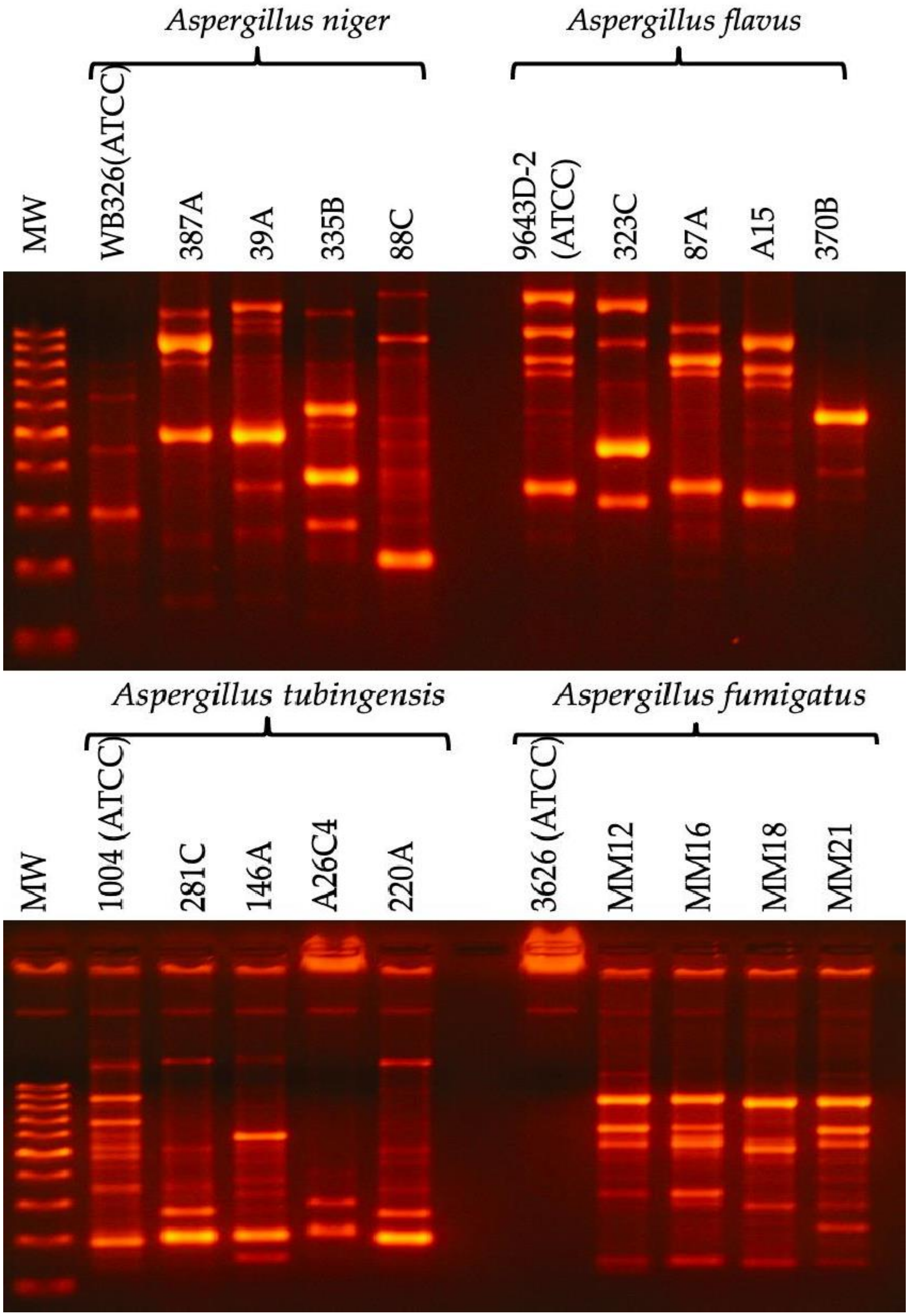


**Figure 12**. Polymorphic patterns obtained by RAPD-PCR for the four species with oligonucleotide R108.

Journal of Fungi

MDPI

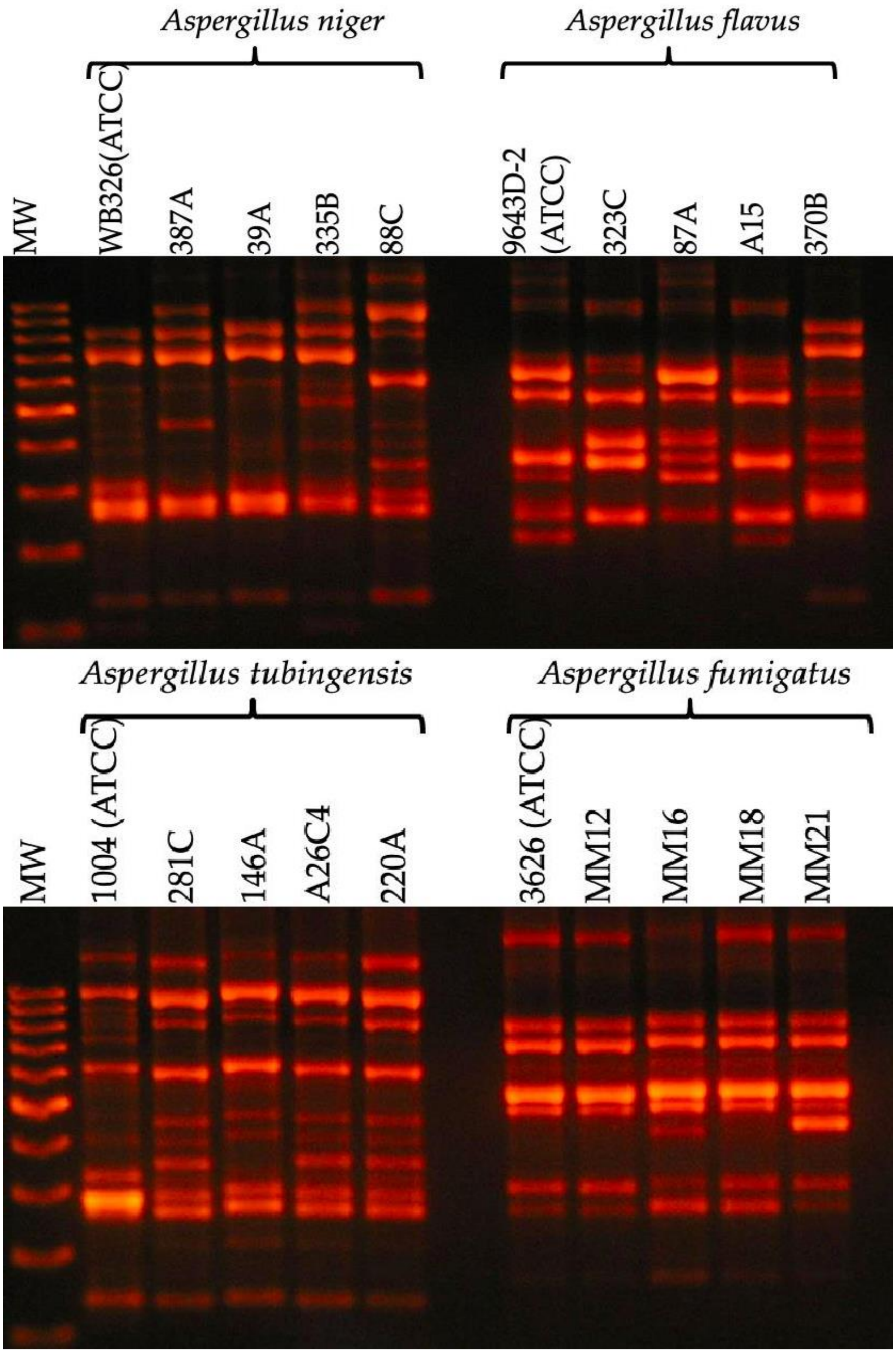


**Figure 13**. Polymorphic patterns obtained by RAPD-PCR for the four species with oligonucleotide 1253.

Journal of Fungi

MDPI

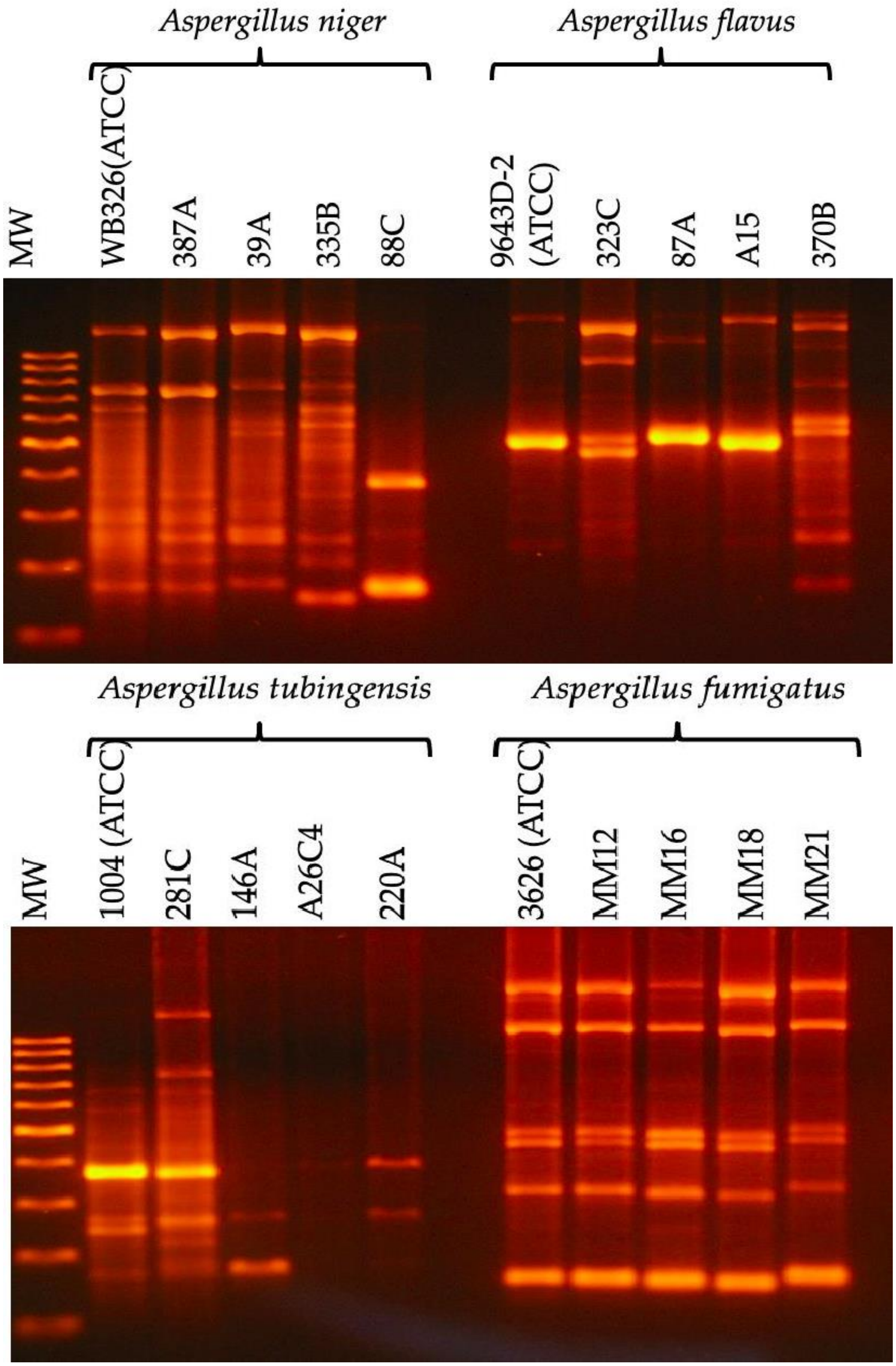


**Figure 14**. Polymorphic patterns obtained by RAPD-PCR for the four species with oligonucleotide OPA-19.

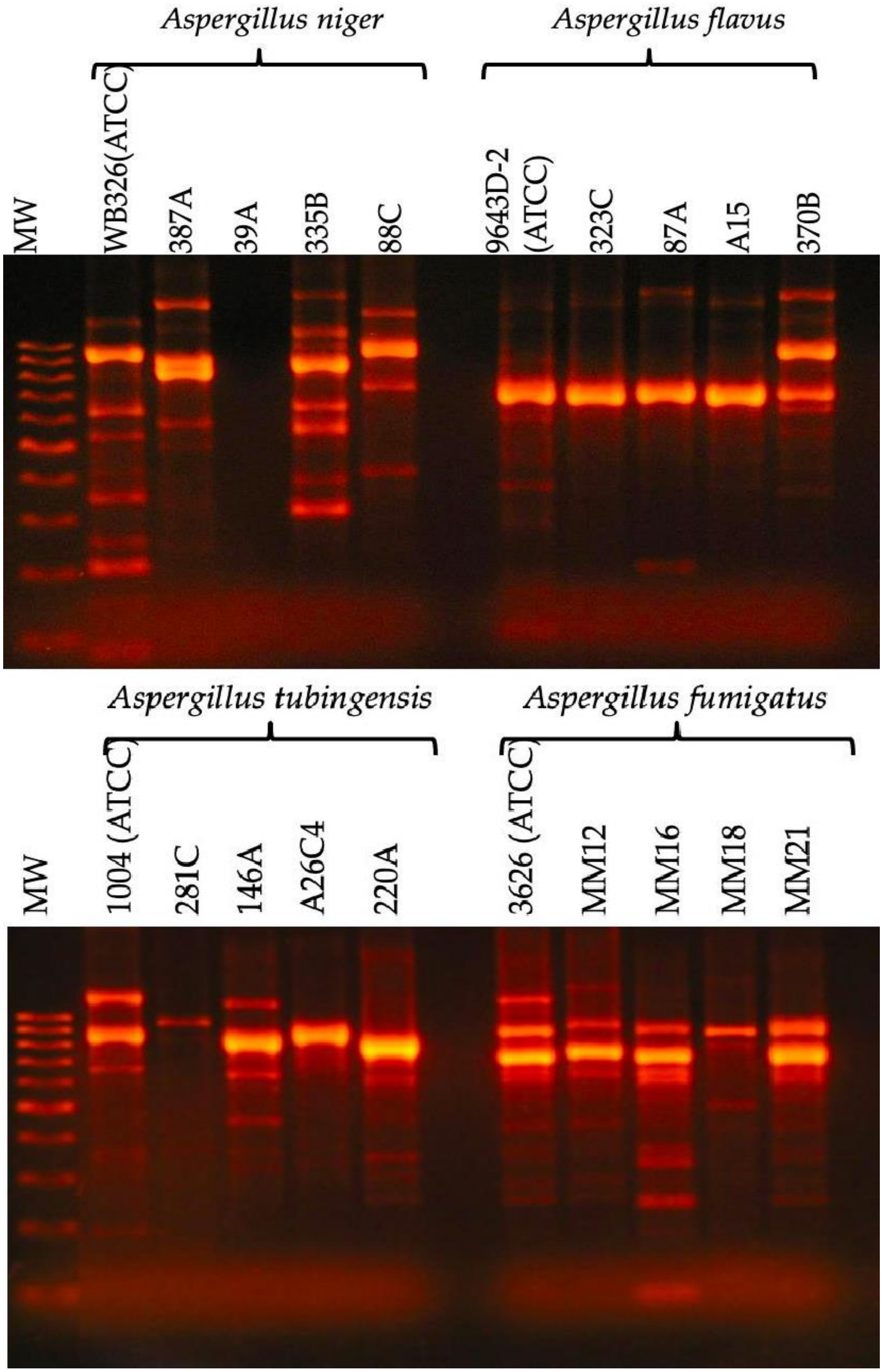


**Figure 15**. Polymorphic patterns obtained by RAPD-PCR for the four species with oligonucleotide OPA-16.

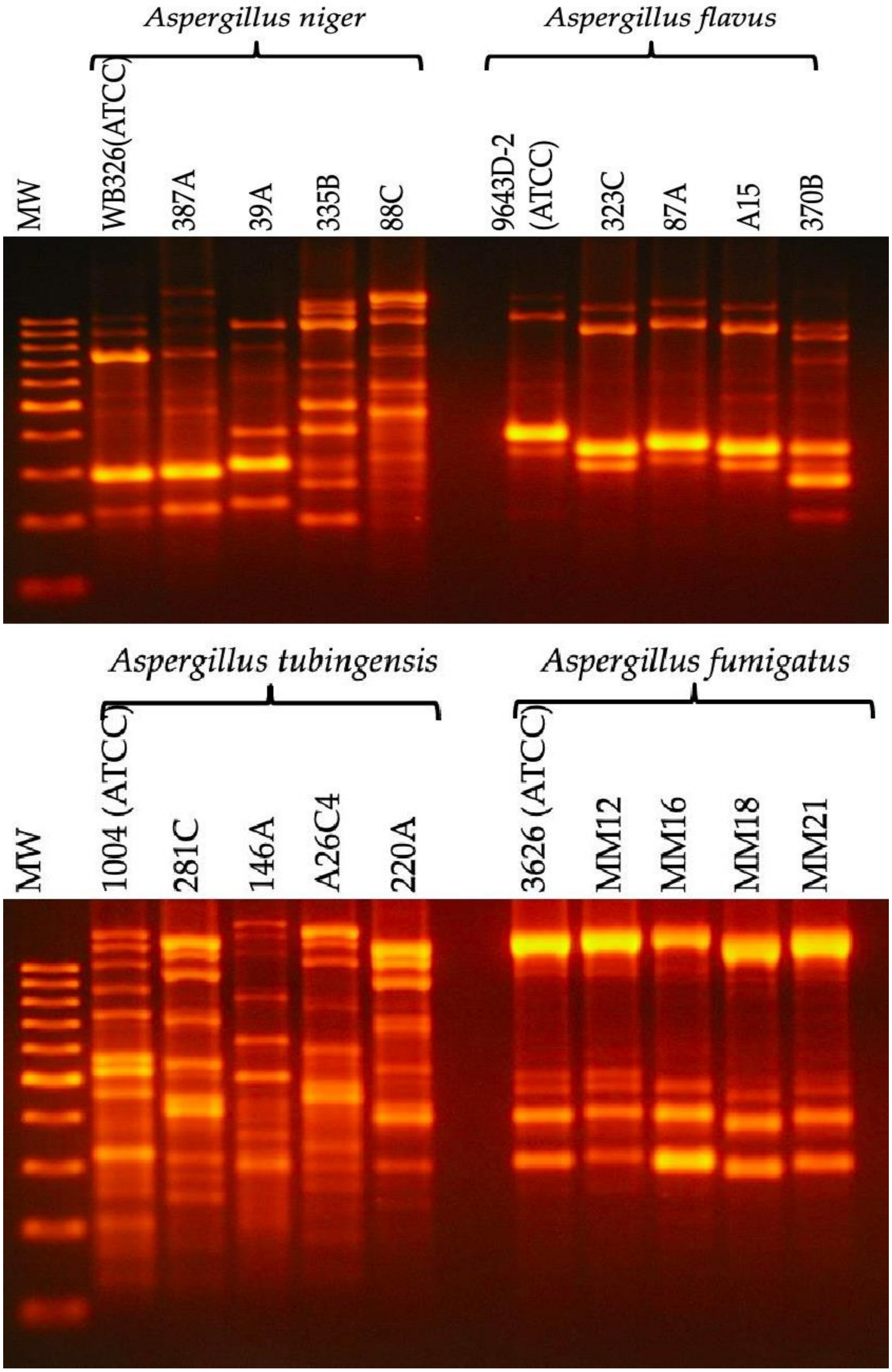


**Figure 16**. Polymorphic patterns obtained by RAPD-PCR for the four species with oligonucleotide OPB-12.

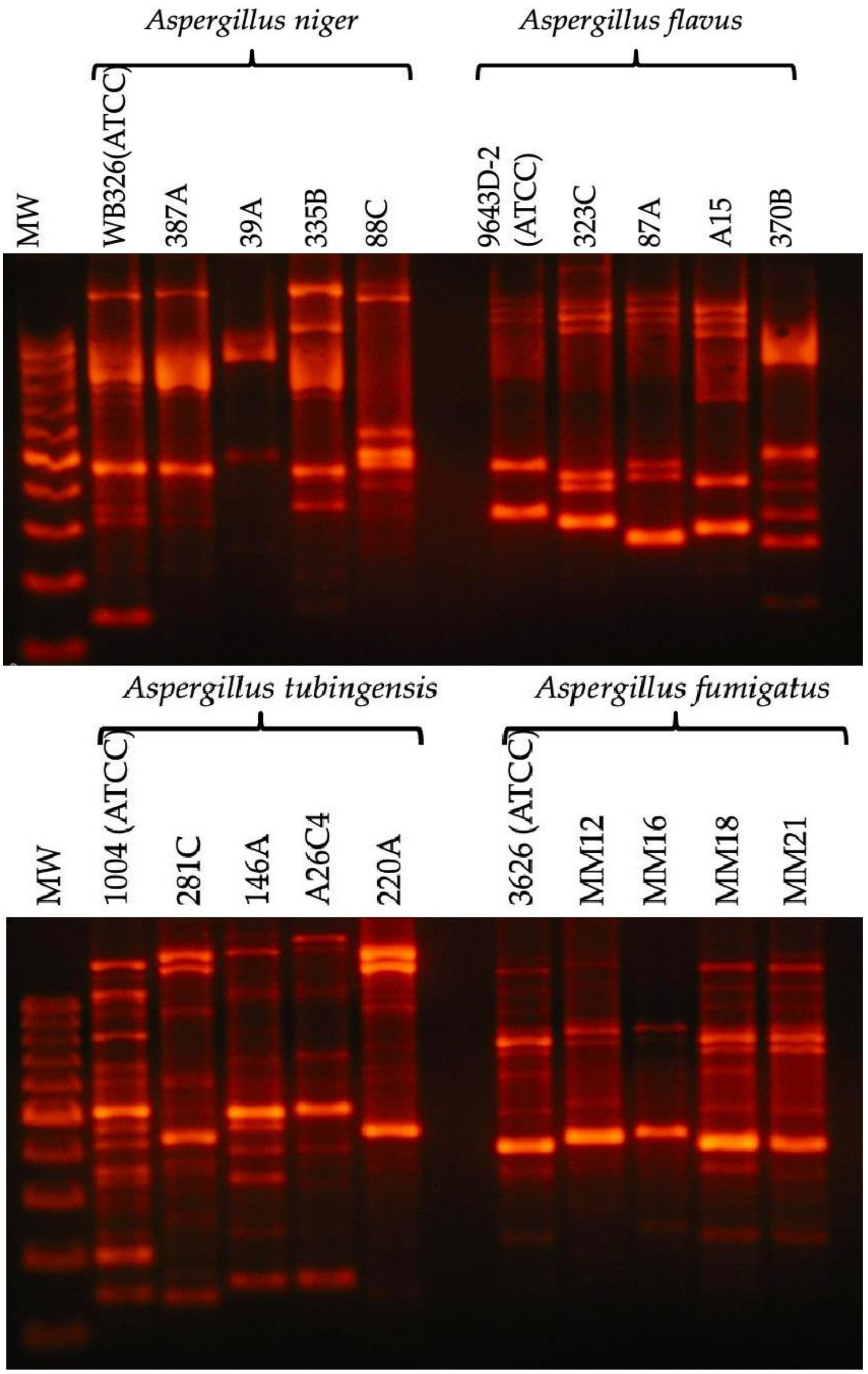


**Figure 17**. Polymorphic patterns obtained by RAPD-PCR for the four species with oligonucleotide OPA-17.

Journal of Fungi

MDPI

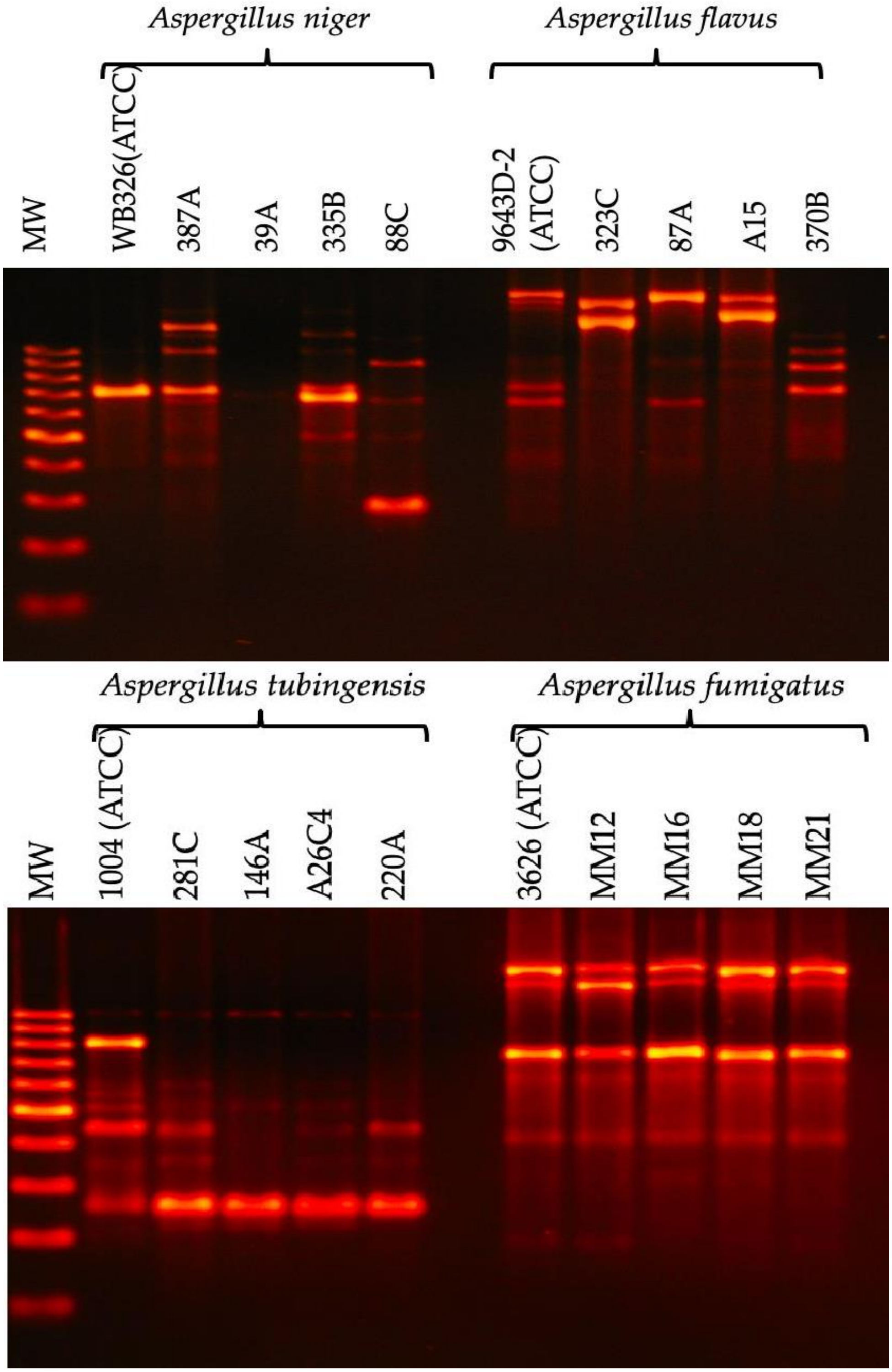


**Figure 18**. Polymorphic patterns obtained by RAPD-PCR for the four species with oligonucleotide OPA-15.

Journal of Fungi

MDPI

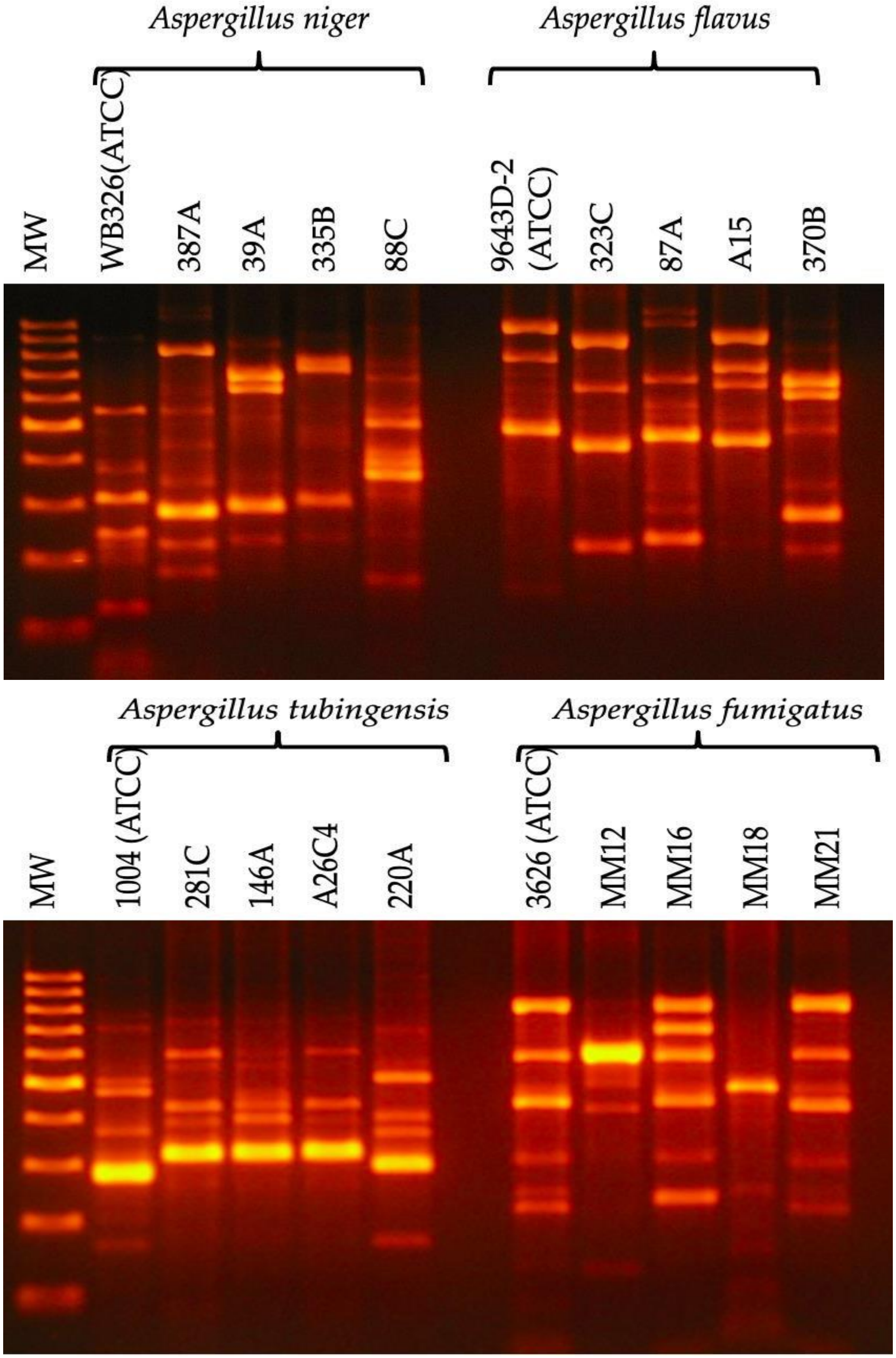


**Figure 19**. Polymorphic patterns obtained by RAPD-PCR for the four species with oligonucleotide OPF-05.

Journal of Fungi

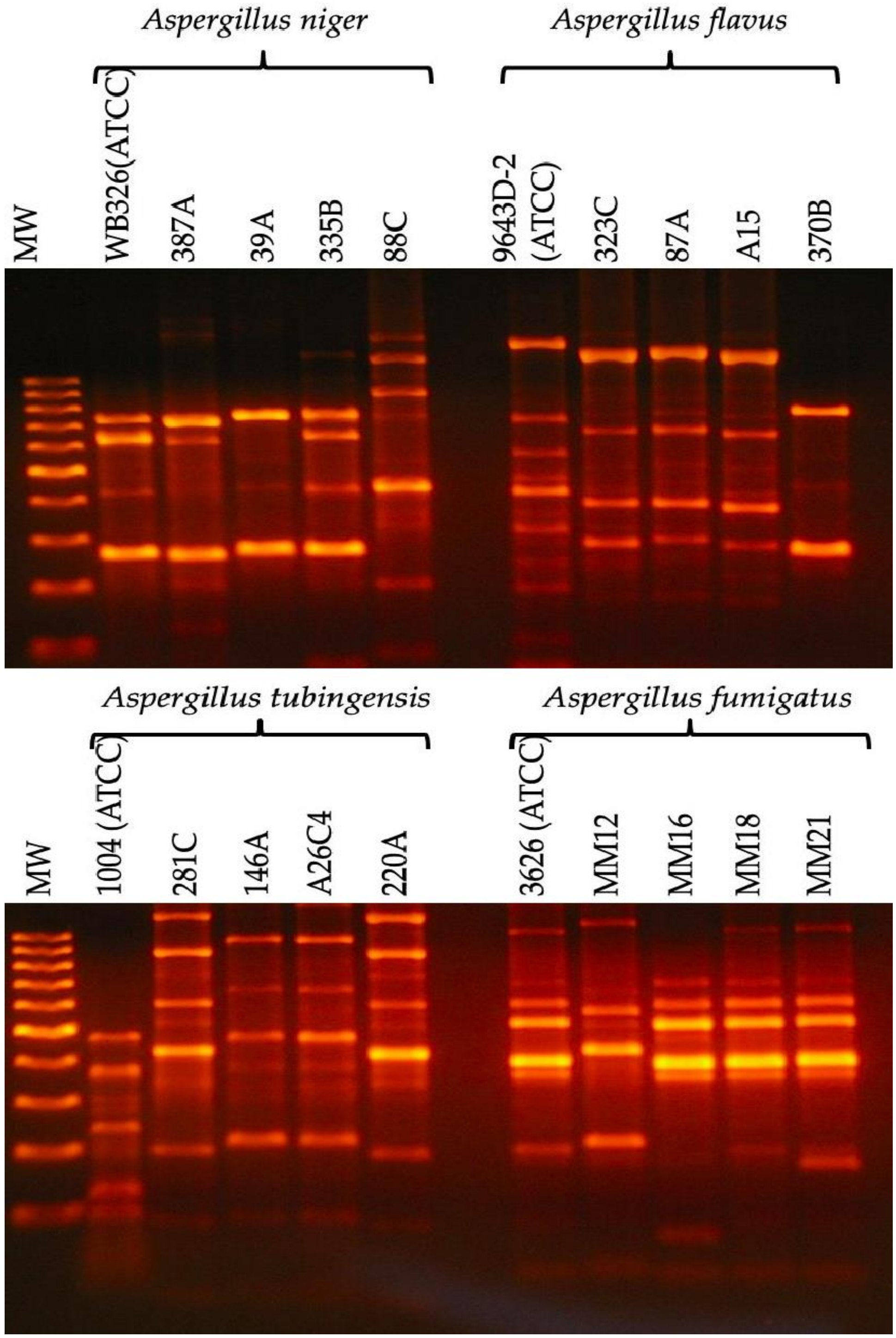


**Figure 20**. Polymorphic patterns obtained by RAPD-PCR for the four species with oligonucleotide OPA-08.

Journal of Fungi

MDPI

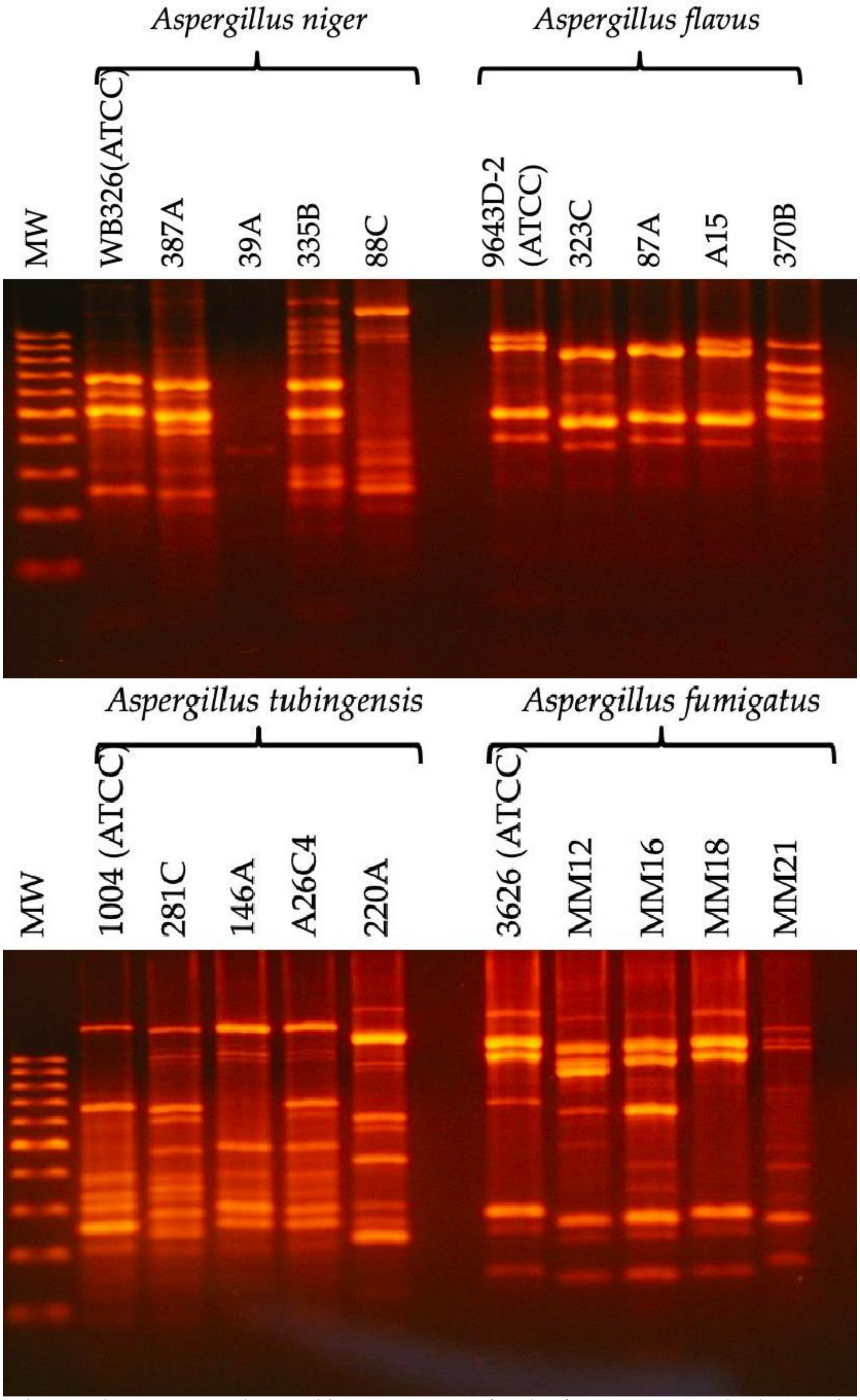


**Figure 21**. Polymorphic patterns obtained by RAPD-PCR for the four species with oligonucleotide OPC-07.

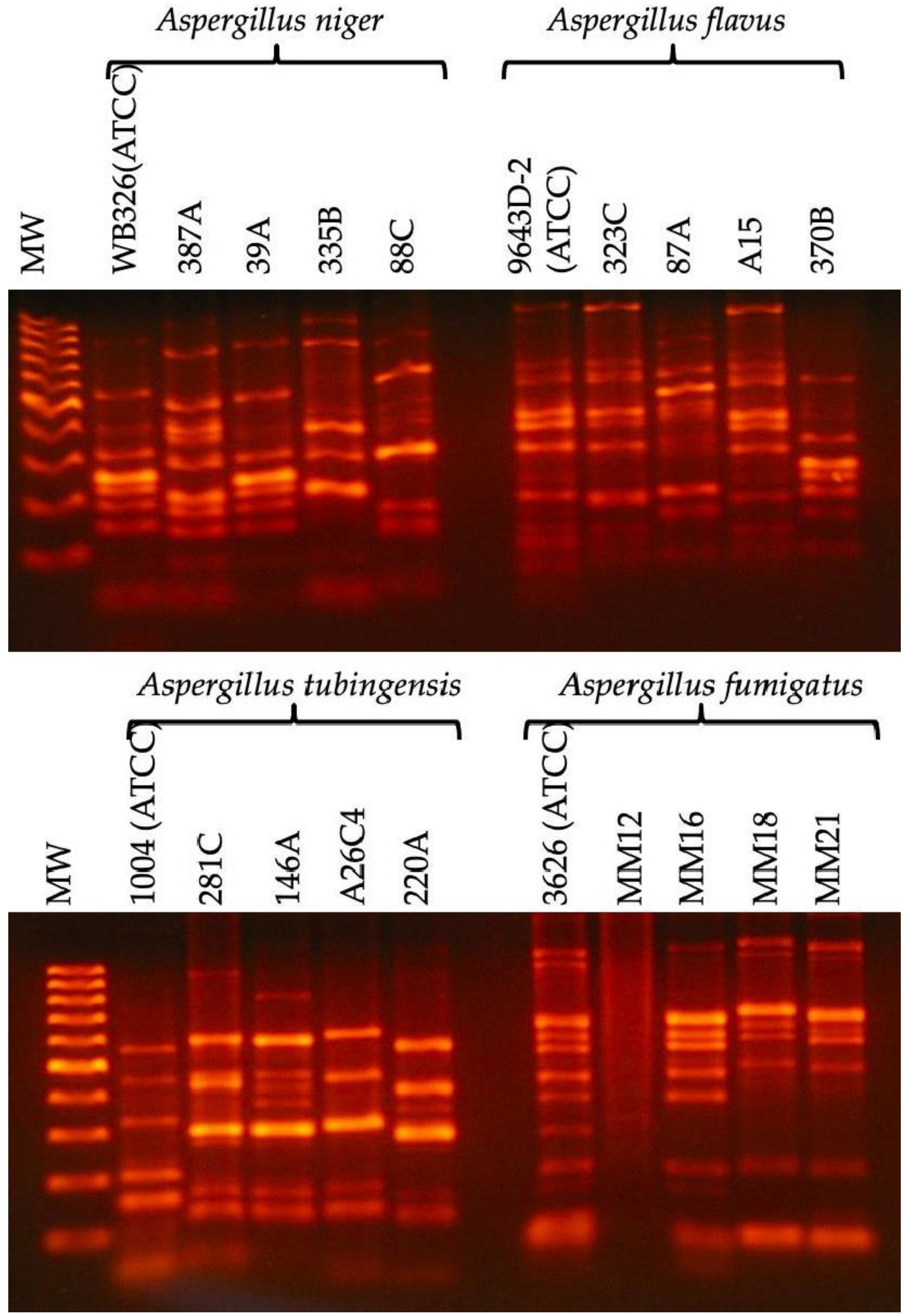


**Figure 22**. Polymorphic patterns obtained by RAPD-PCR for the four species with oligonucleotide OPG-01.

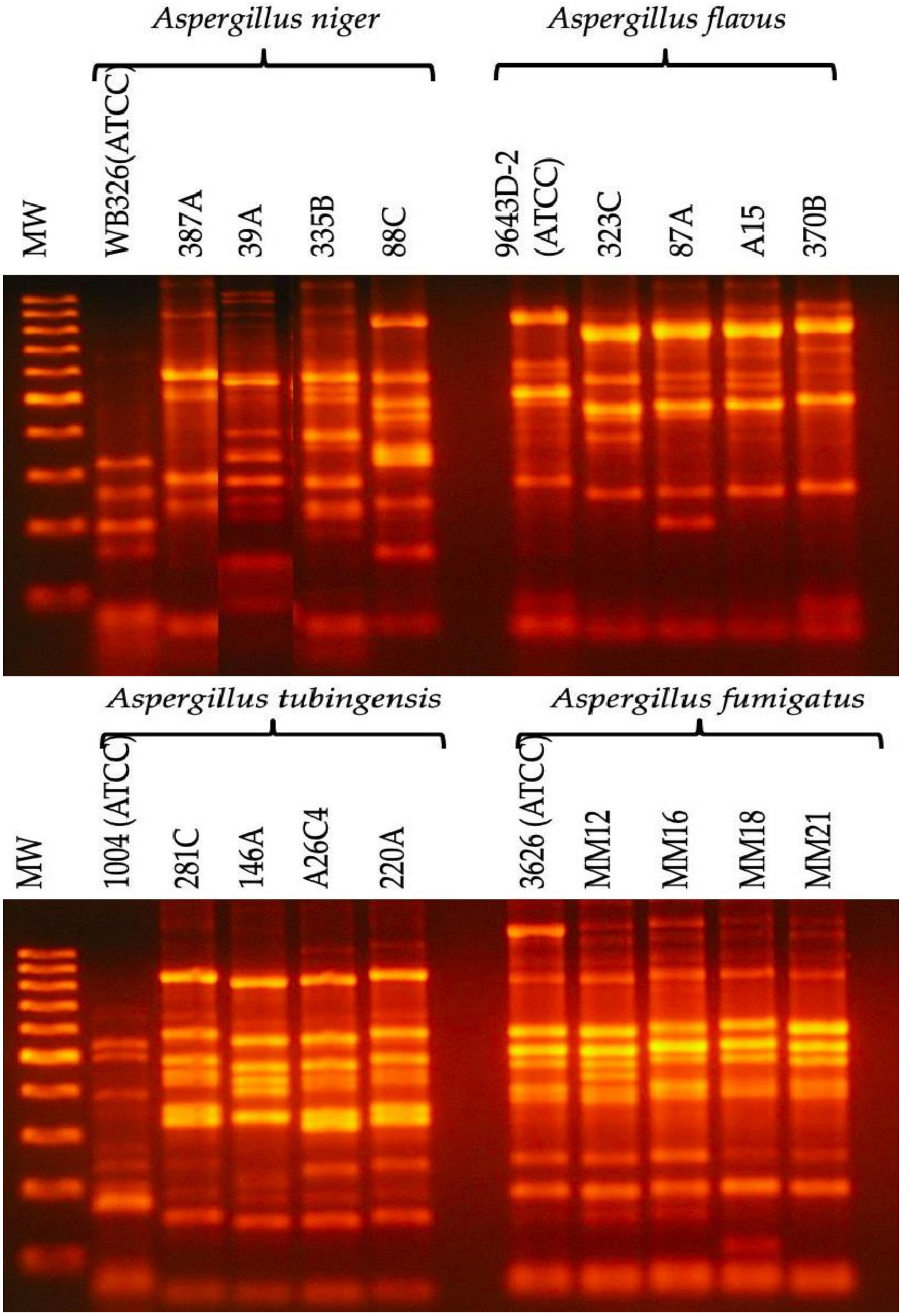


**Figure 23**. Polymorphic patterns obtained by RAPD-PCR for the four species with oligonucleotide OPG-03.

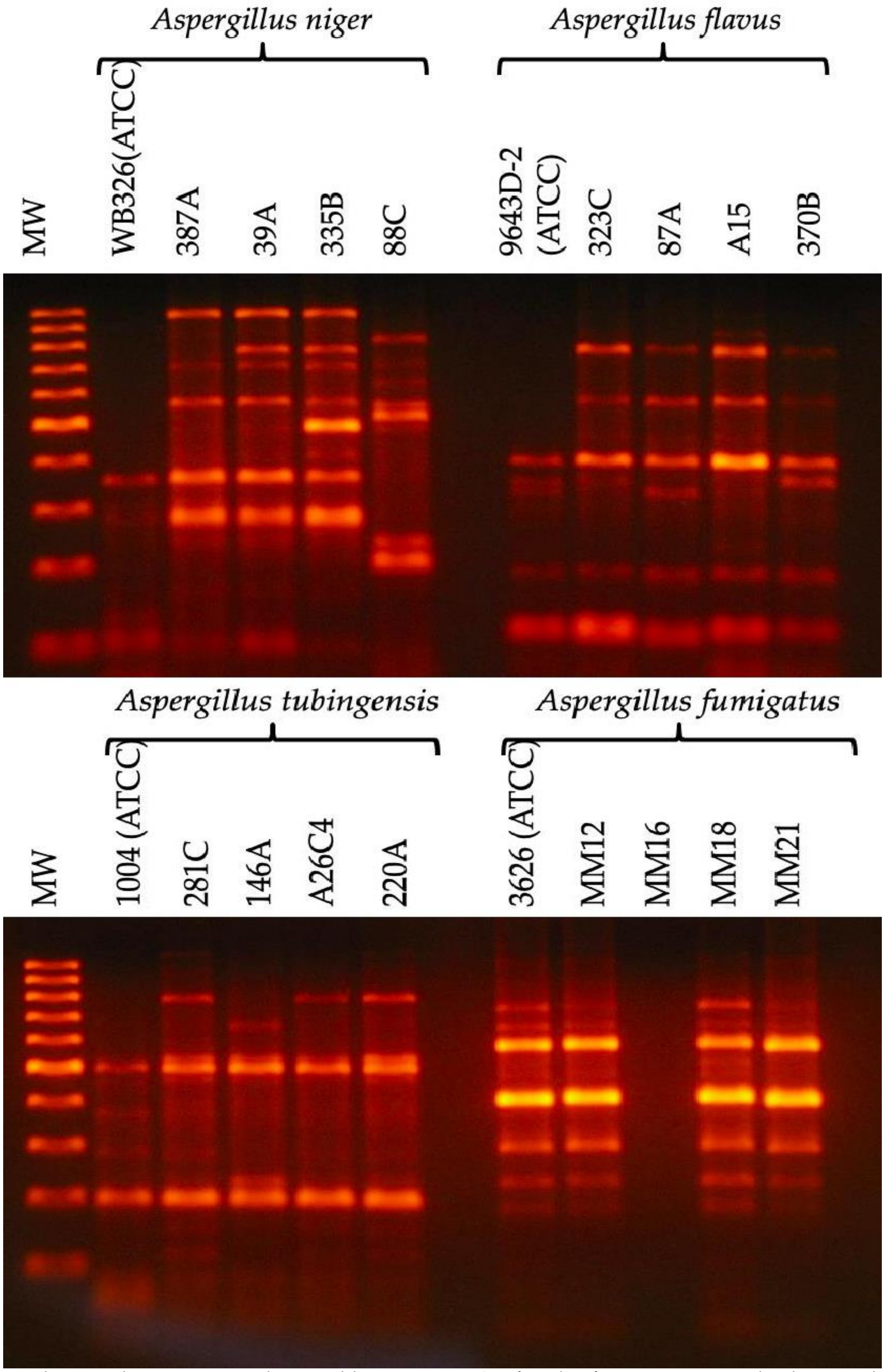


**Figure 24**. Polymorphic patterns obtained by RAPD-PCR for the four species with oligonucleotide OPG-13.

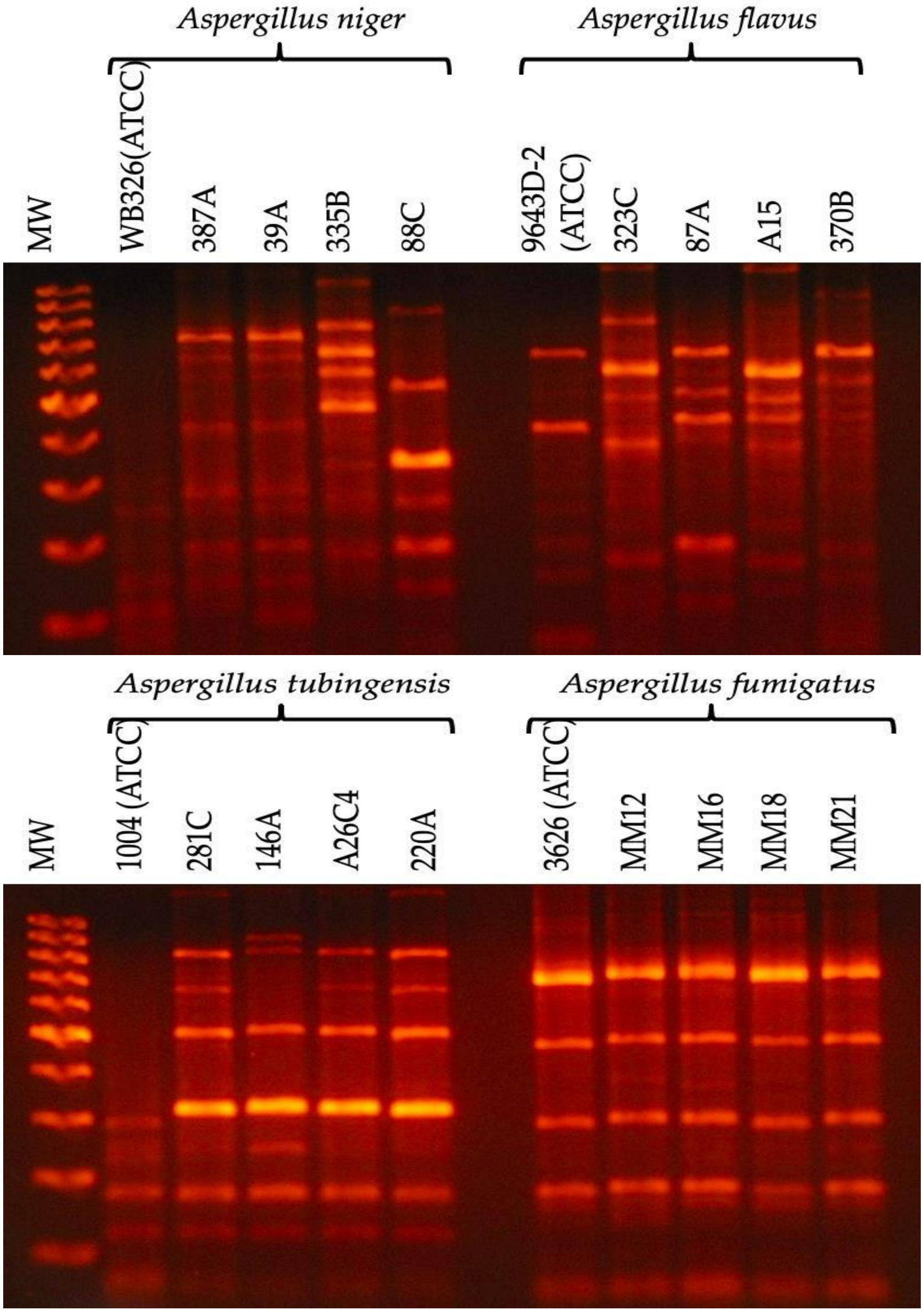


**Figure 25**. Polymorphic patterns obtained by RAPD-PCR for the four species with oligonucleotide OPG-15.

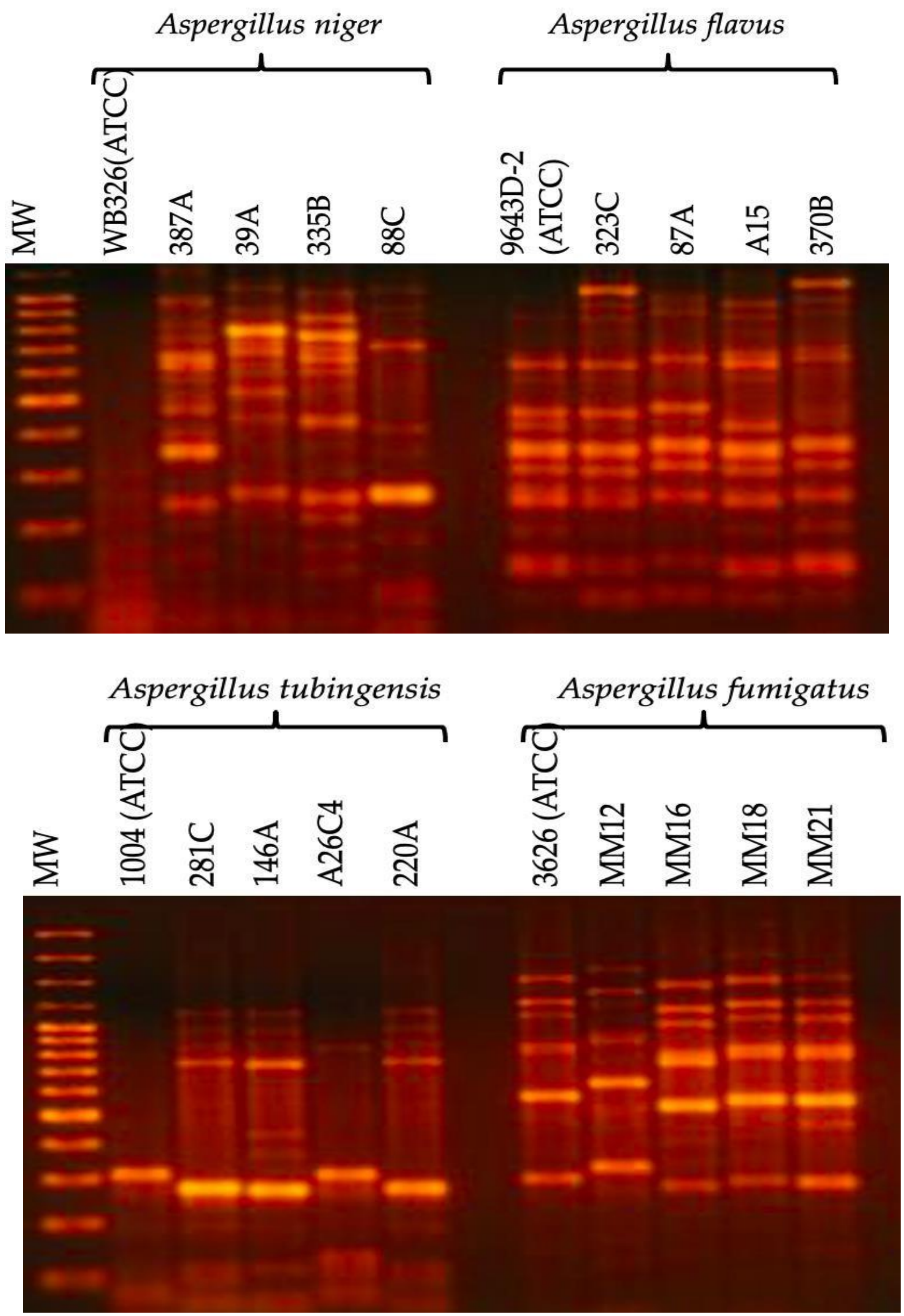


**Figure 26**. Polymorphic patterns obtained by RAPD-PCR for the four species with oligonucleotide OPC-06.

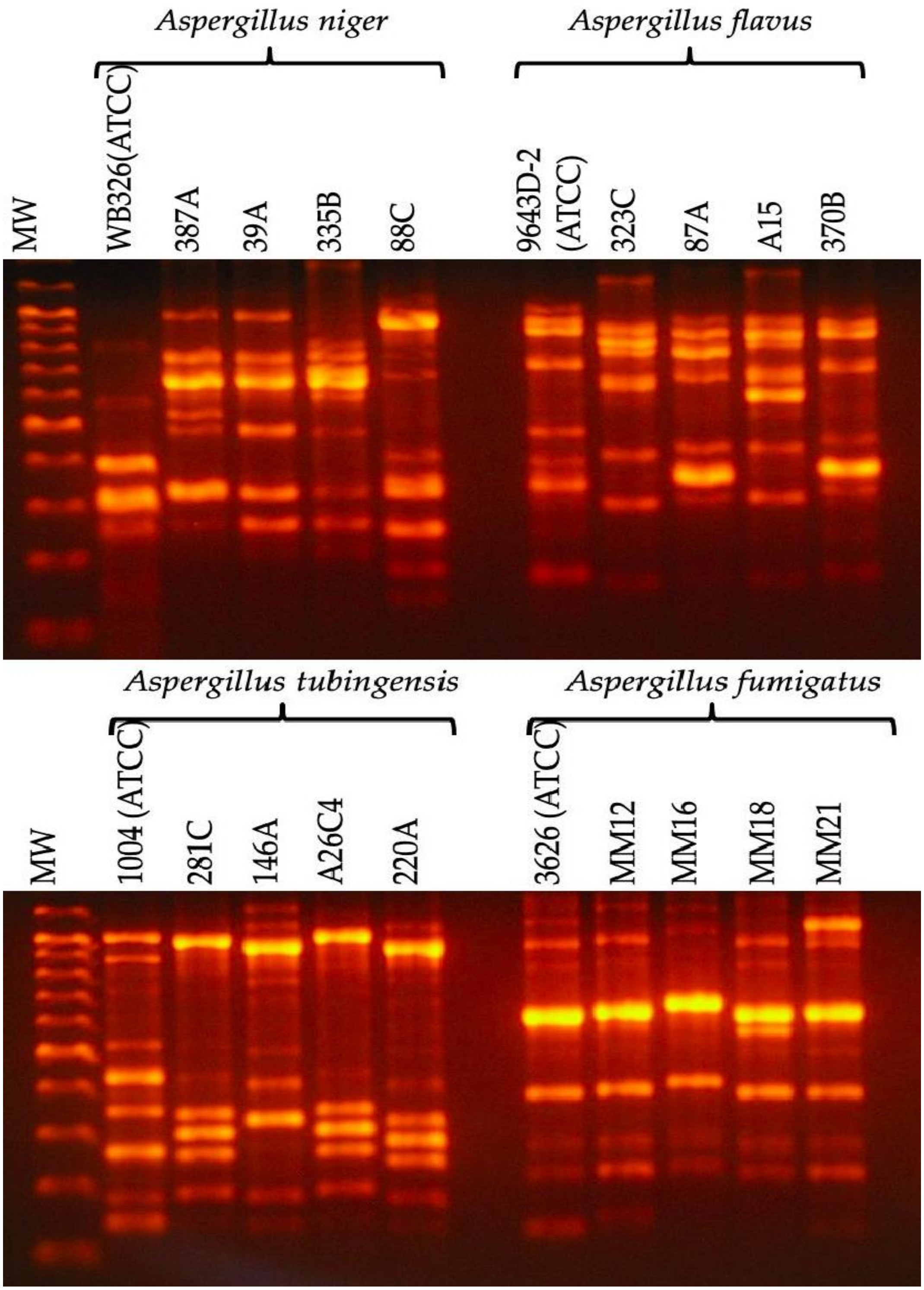


**Figure 27**. Polymorphic patterns obtained by RAPD-PCR for the four species with oligonucleotide OPG-05.

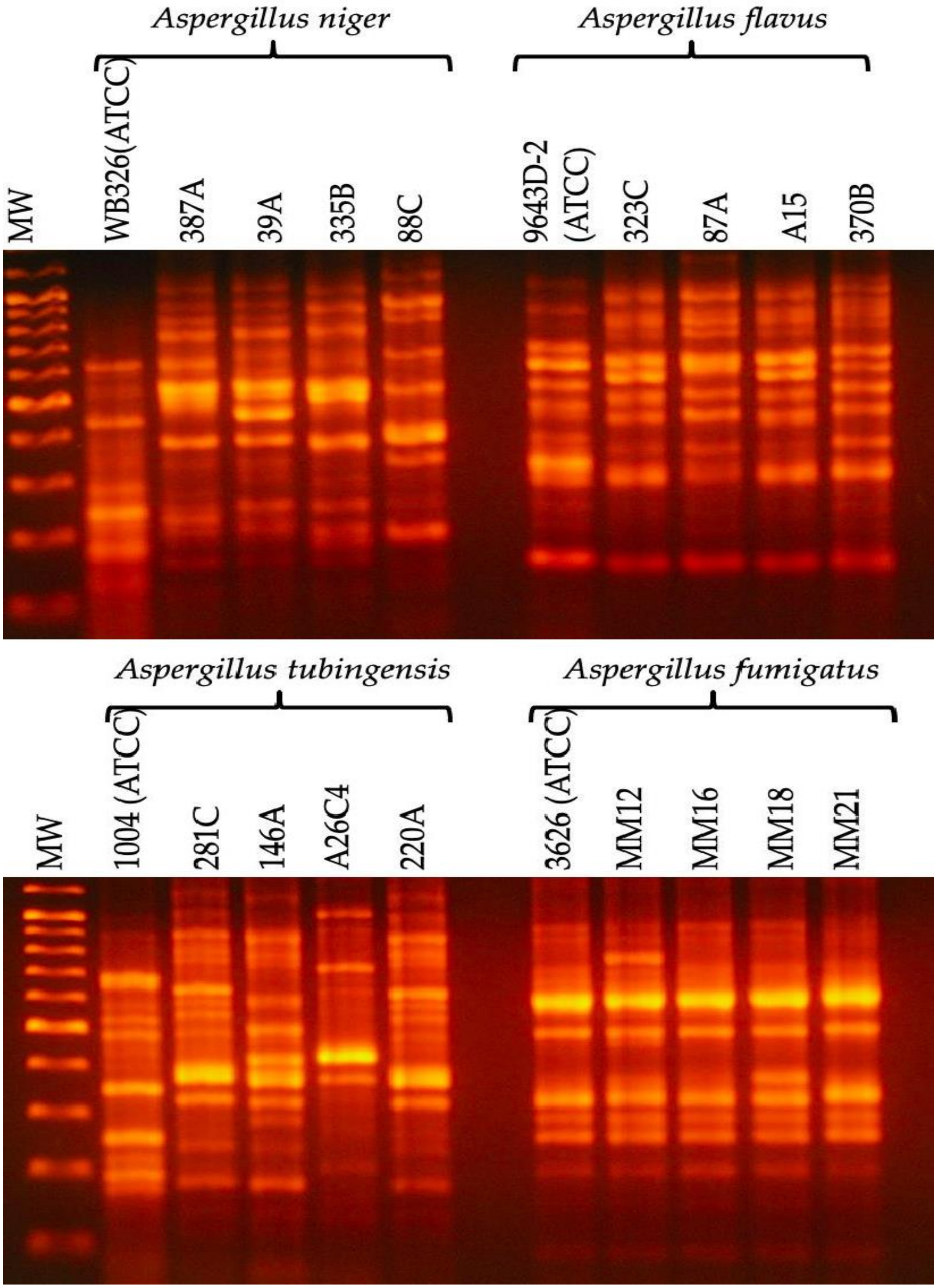


**Figure 28**. Polymorphic patterns obtained by RAPD-PCR for the four species with oligonucleotide OPM-12.

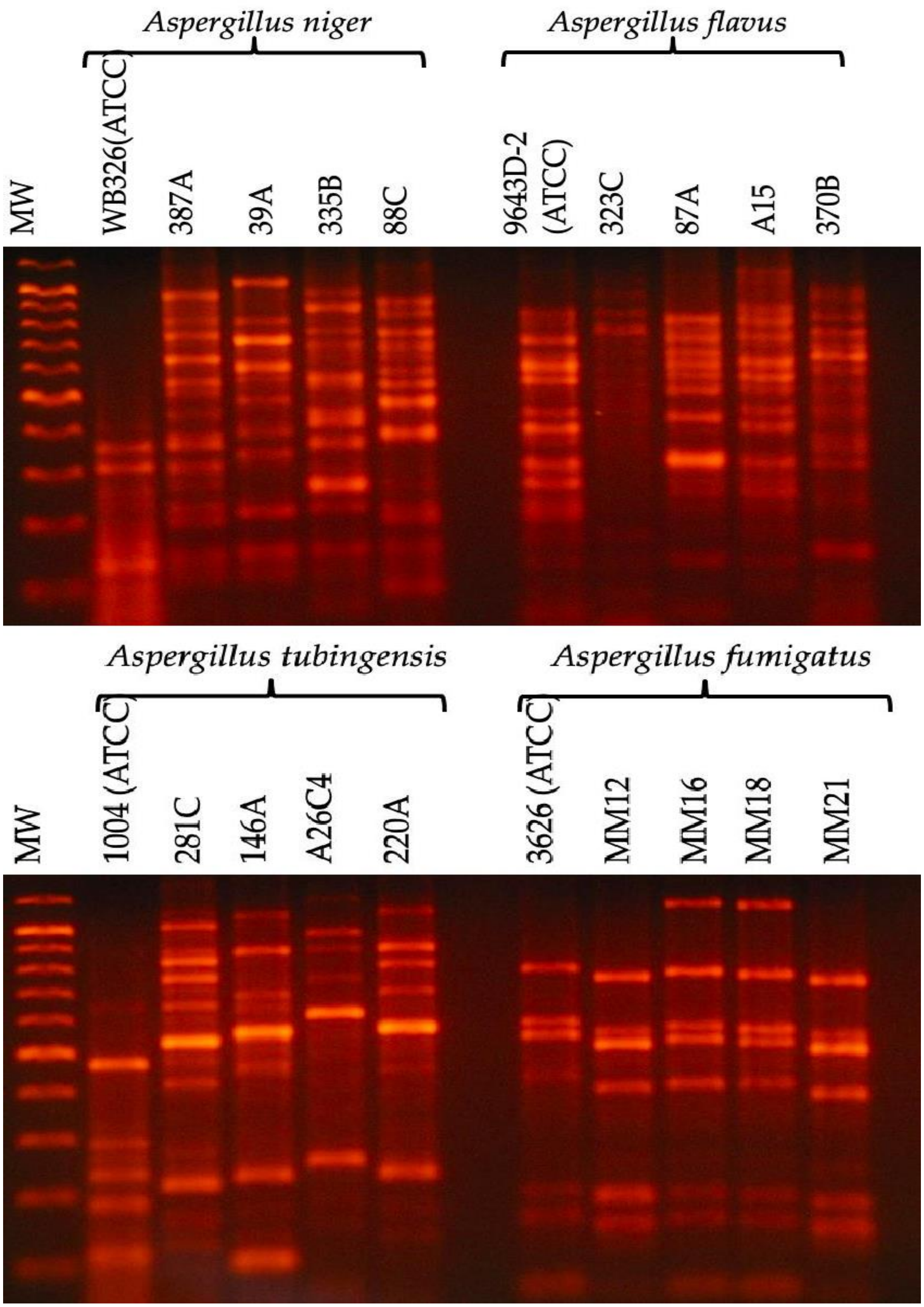


**Figure 29**. Polymorphic patterns obtained by RAPD-PCR for the four species with oligonucleotide OPH-03.

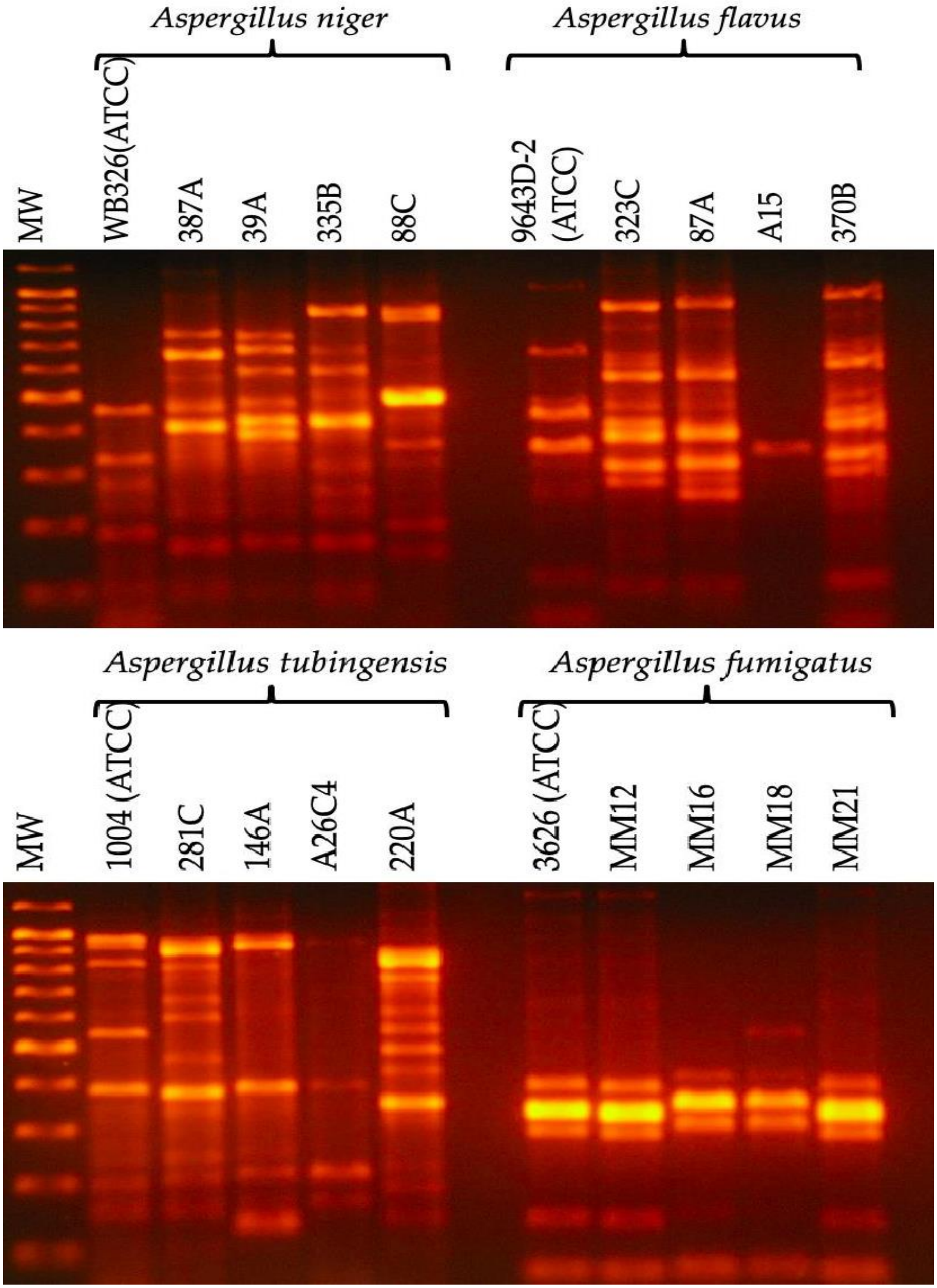


**Figure 30**. Polymorphic patterns obtained by RAPD-PCR for the four species with oligonucleotide OPG-07.

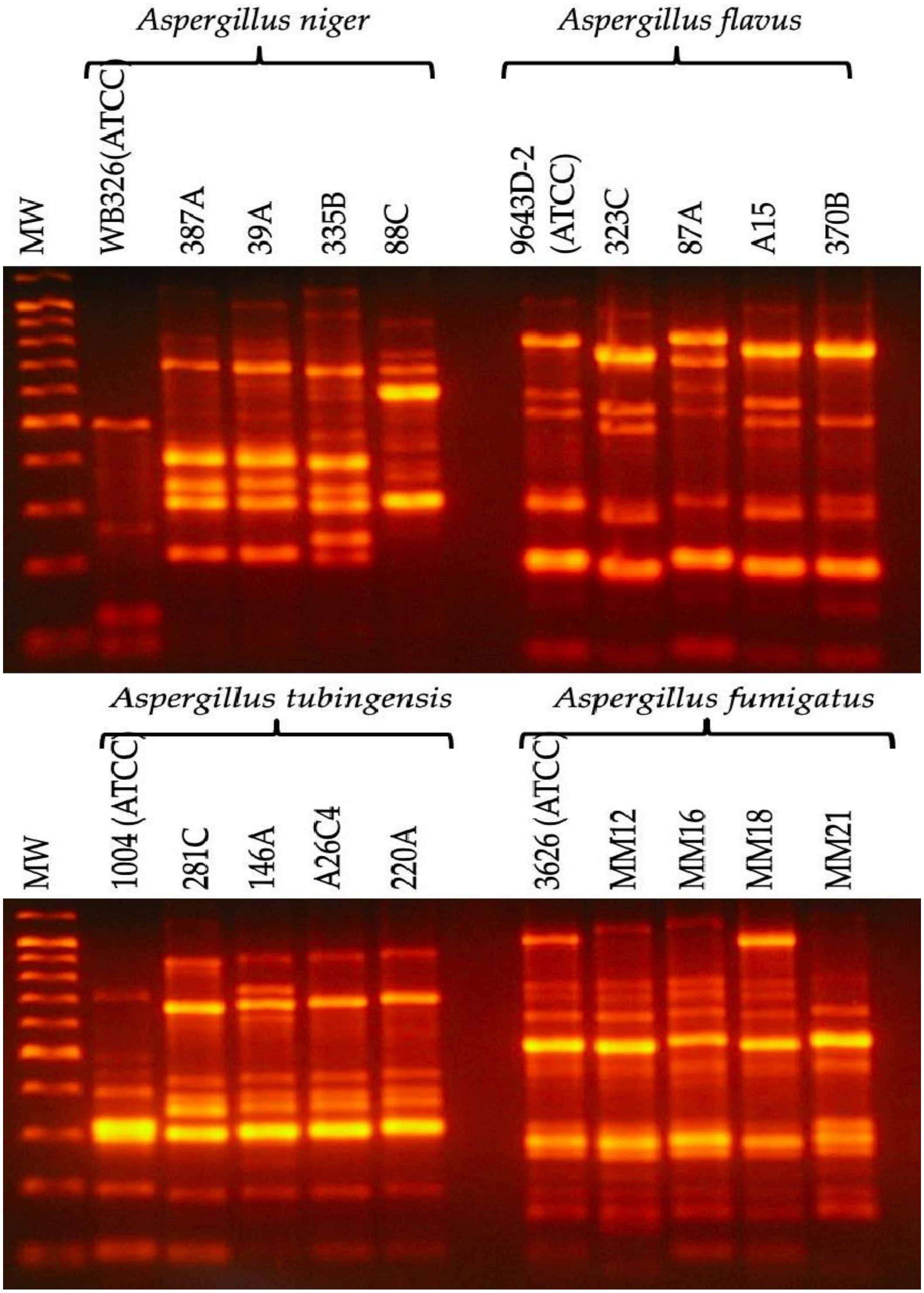


**Figure 31**. Polymorphic patterns obtained by RAPD-PCR for the four species with oligonucleotide OPH-18.

Journal of Fungi

MDPI

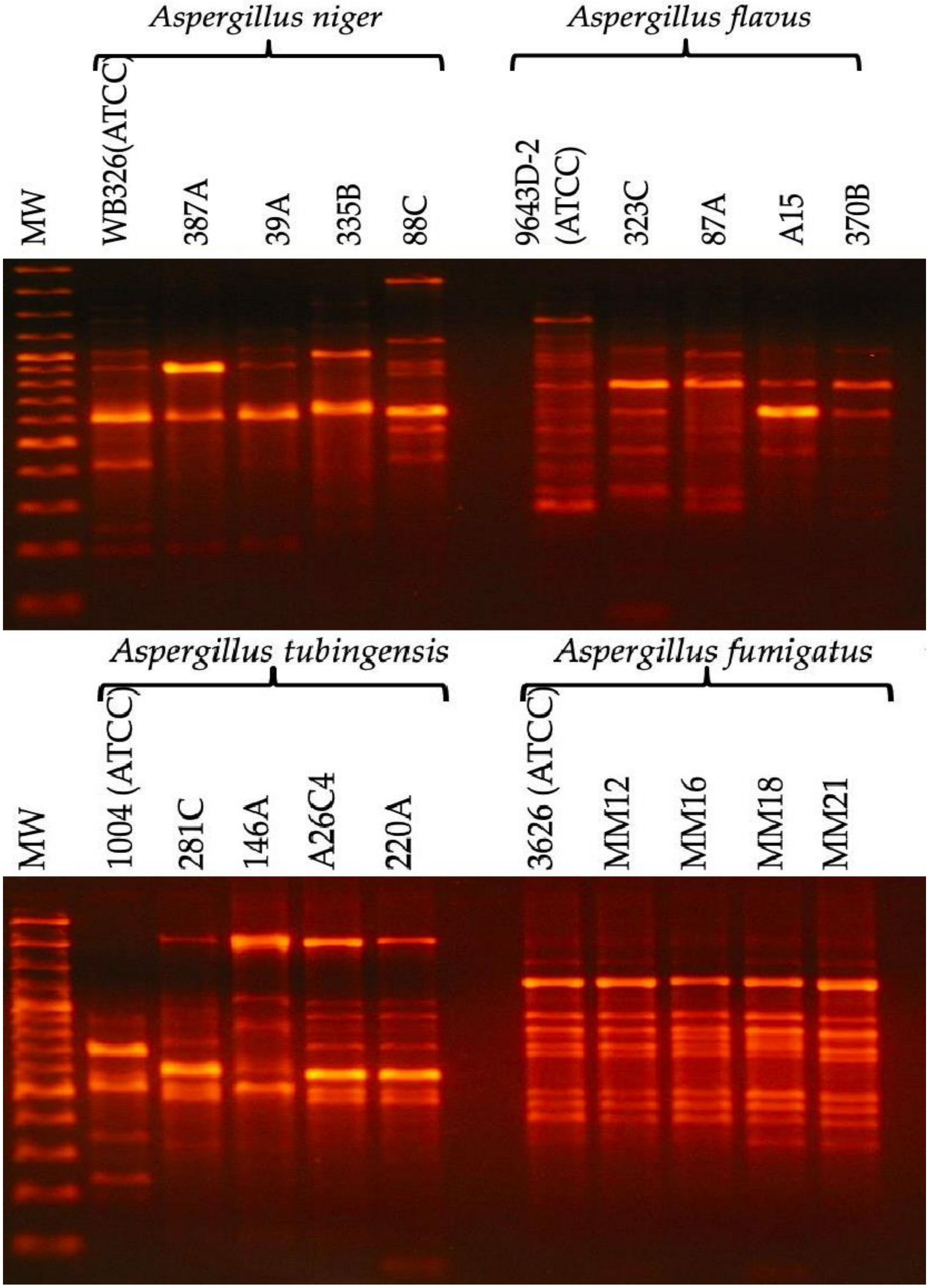


**Figure 32**. Polymorphic patterns obtained by RAPD-PCR for the four species with oligonucleotide OPF-01.

Journal of Fungi

MDPI

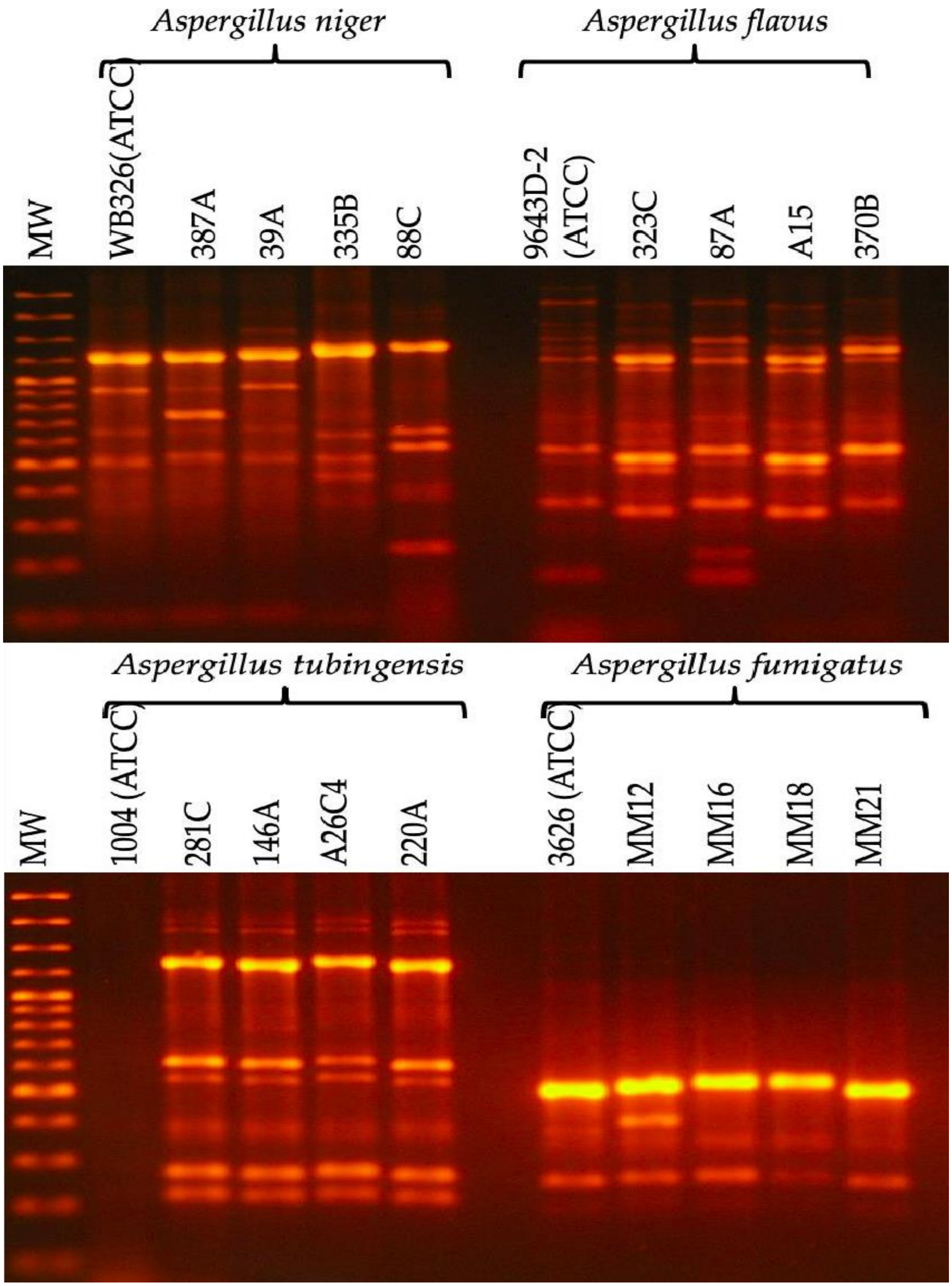


**Figure 33**. Polymorphic patterns obtained by RAPD-PCR for the four species with oligonucleotide OPF-07.

Journal of Fungi

MDPI

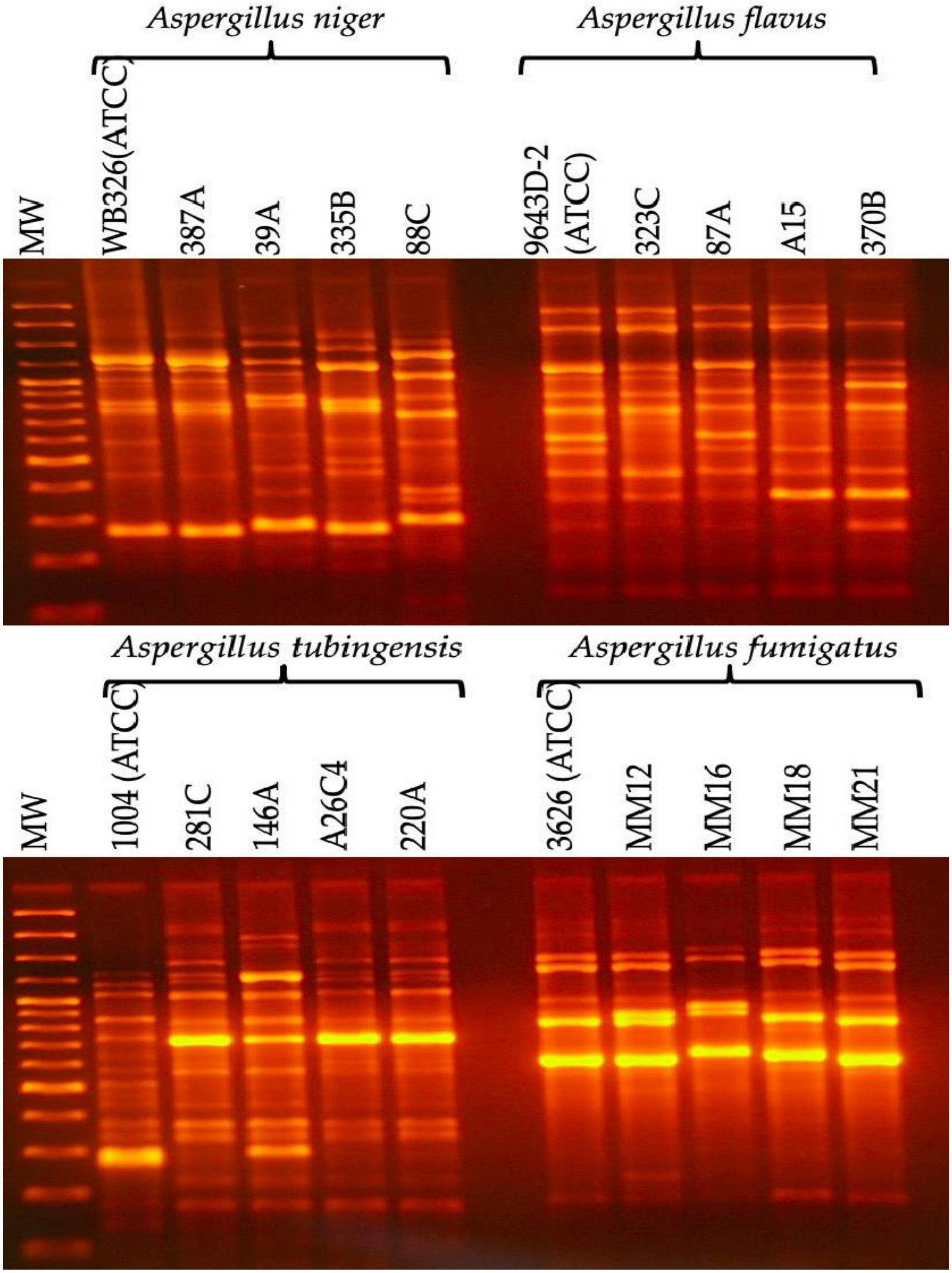


**Figure 34**. Polymorphic patterns obtained by RAPD-PCR for the four species with oligonucleotide OPF-09.

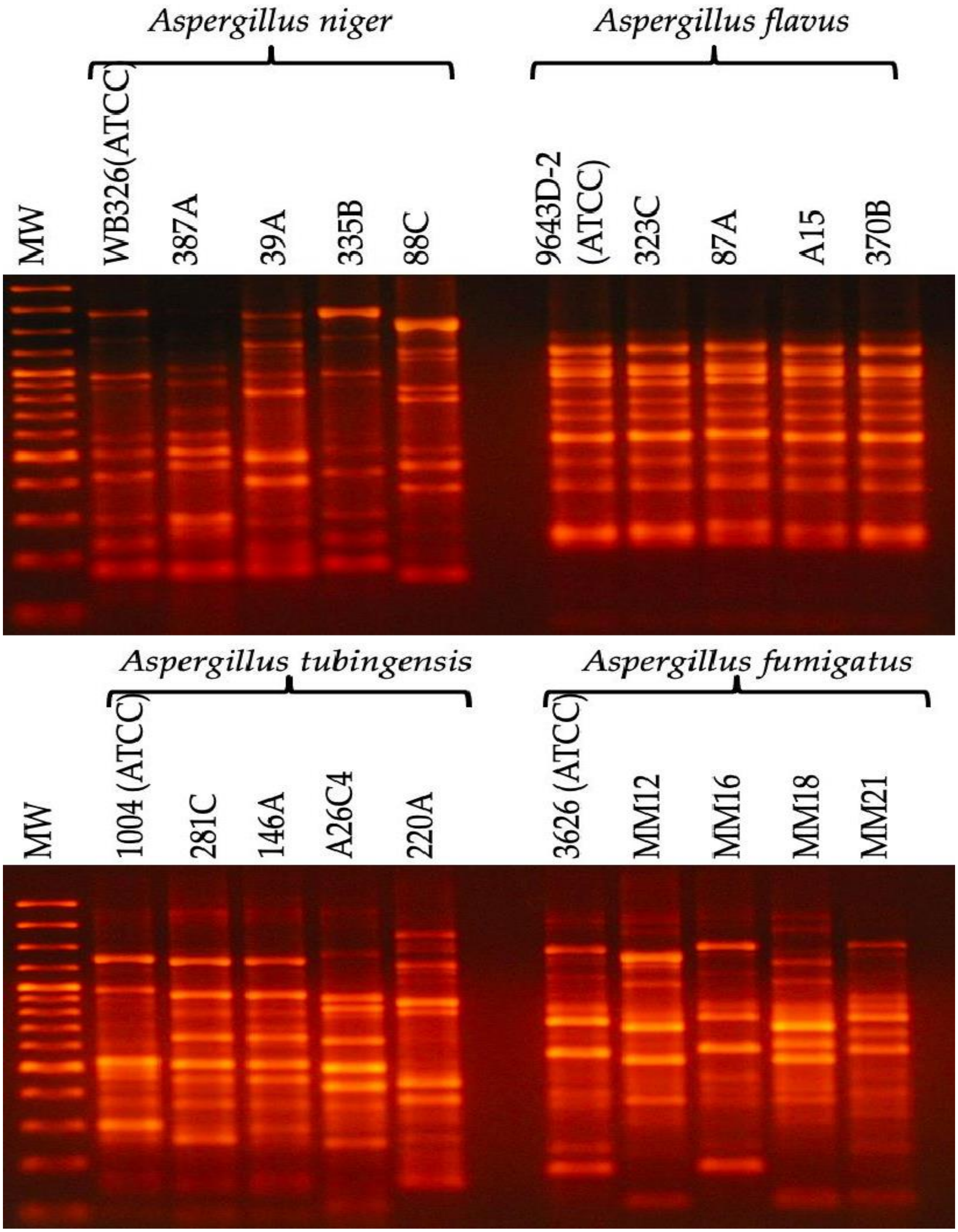


**Figure 35**. Polymorphic patterns obtained by RAPD-PCR for the four species with oligonucleotide OPE-02.

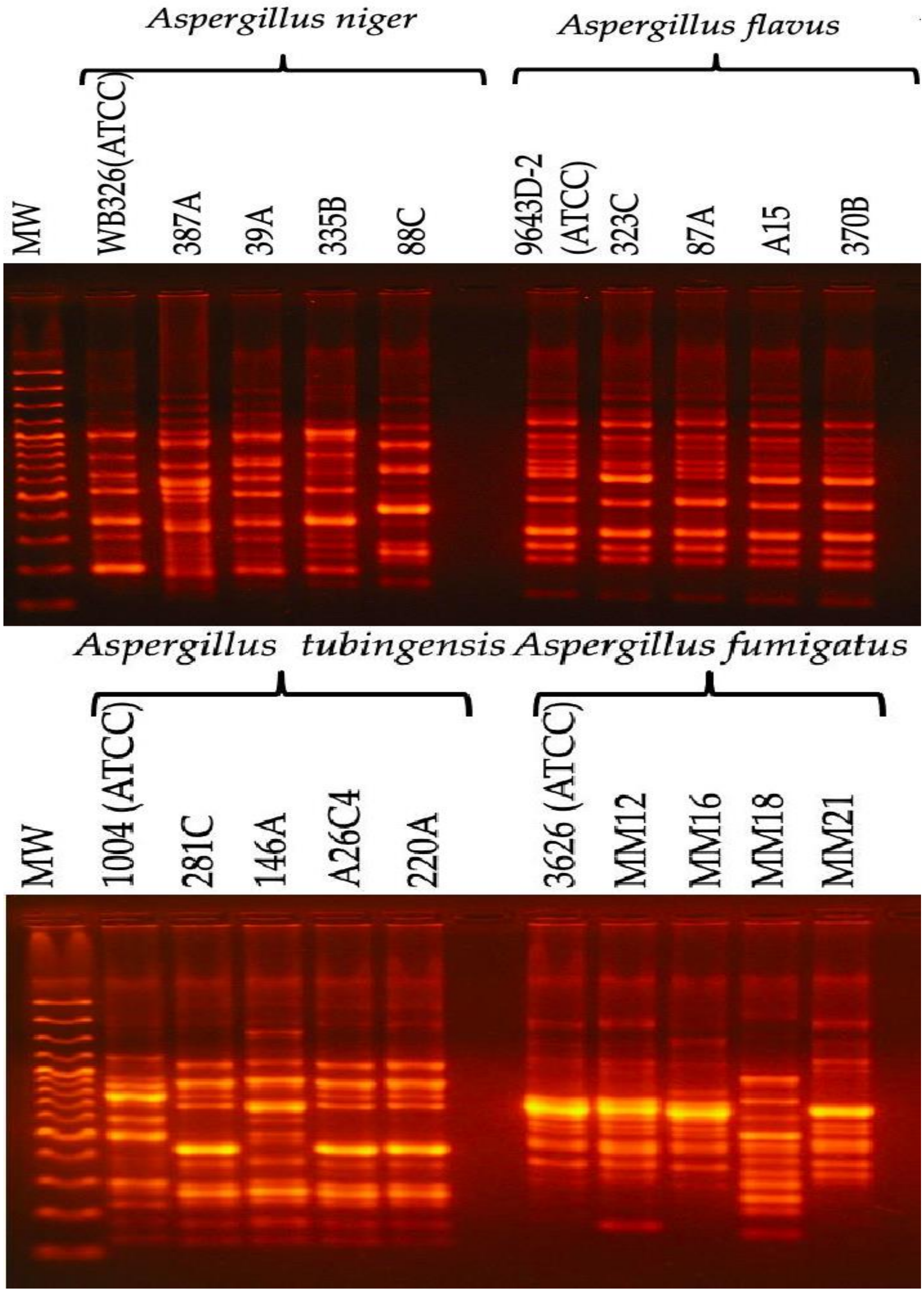


**Figure 36**. Polymorphic patterns obtained by RAPD-PCR for the four species with oligonucleotide P54.

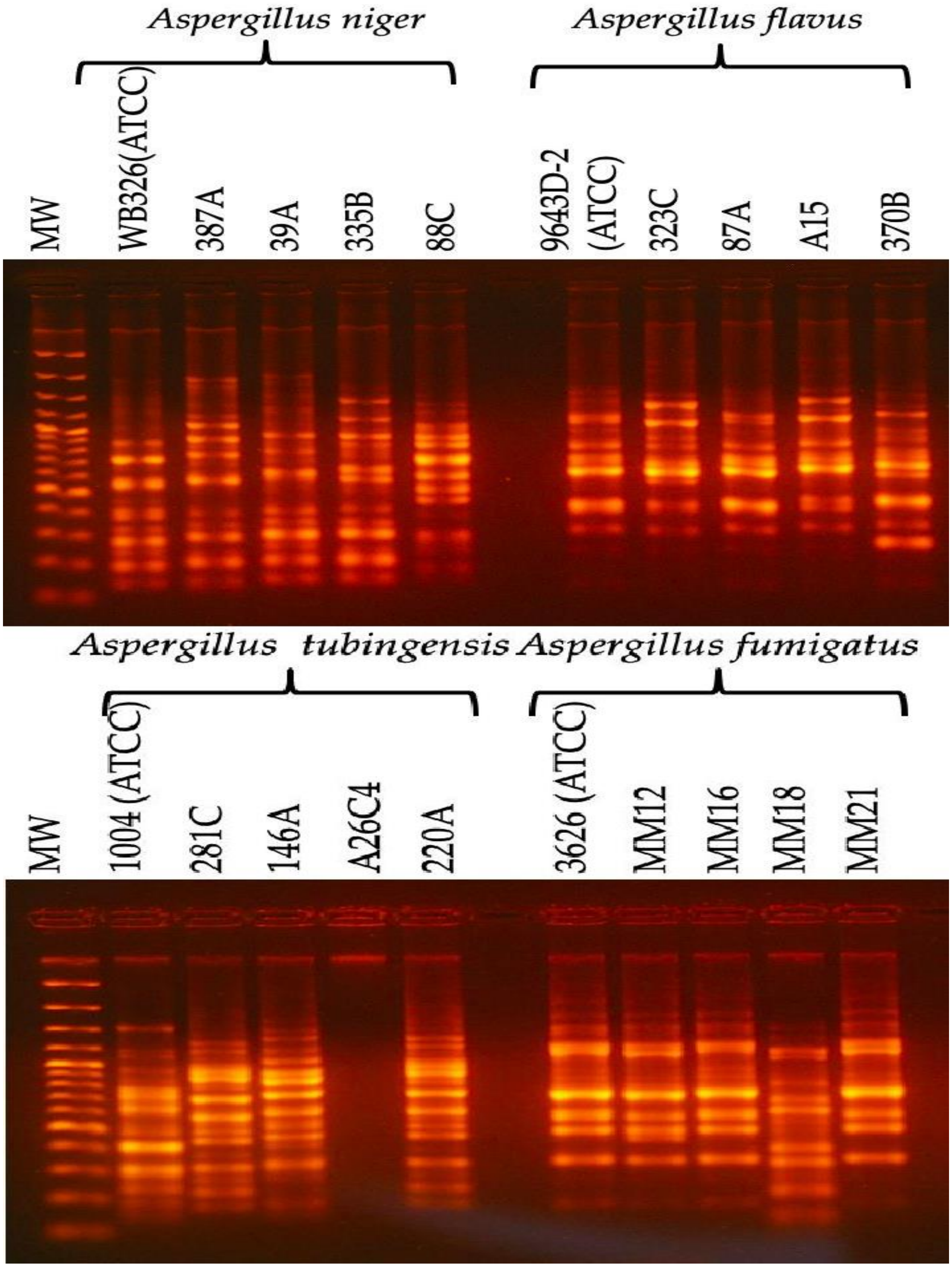


**Figure 37**. Polymorphic patterns obtained by RAPD-PCR for the four species with oligonucleotide P160.

Journal of Fungi

MDPI

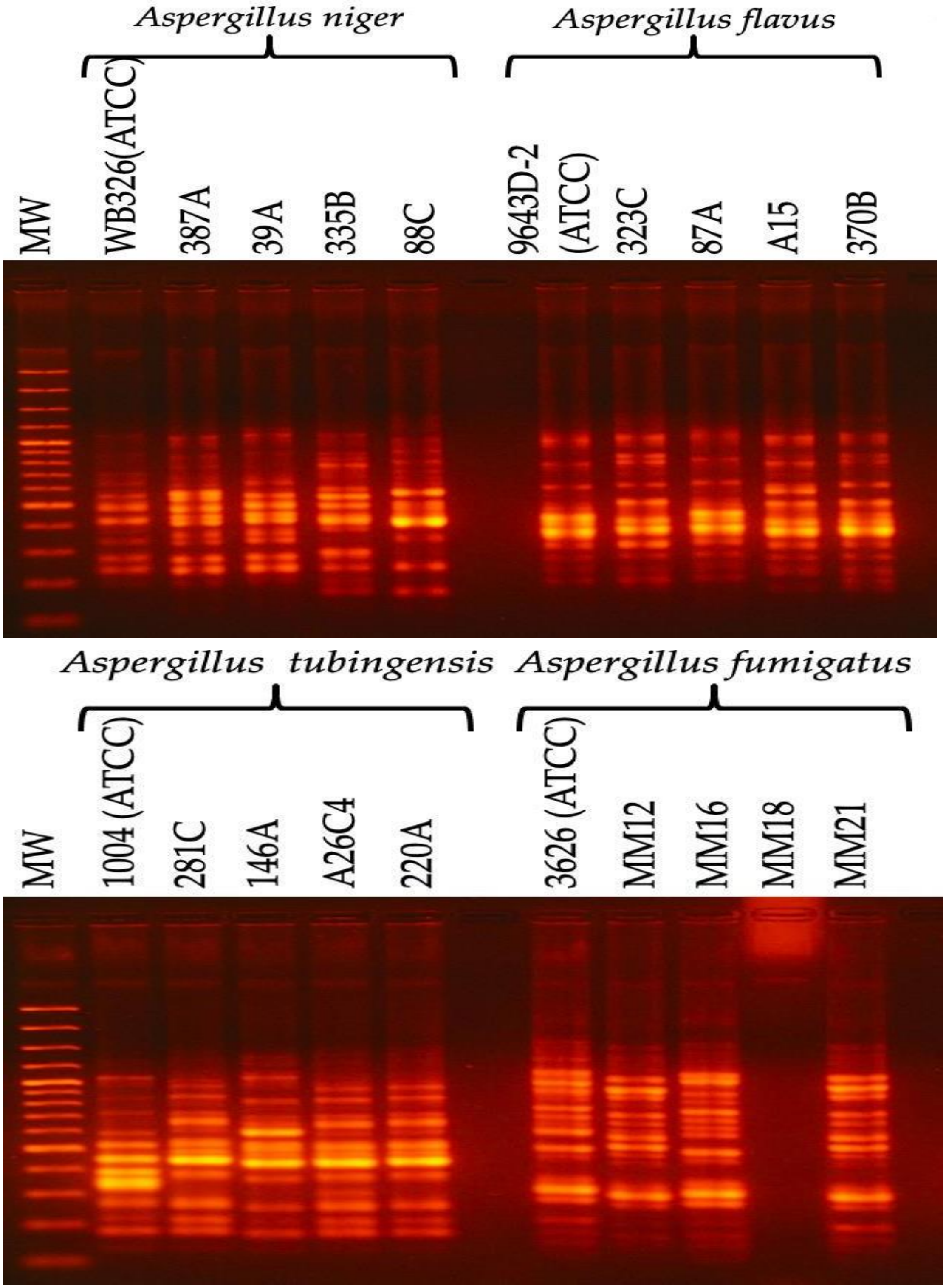


**Figure 38**. Polymorphic patterns obtained by RAPD-PCR for the four species with oligonucleotide PELF.

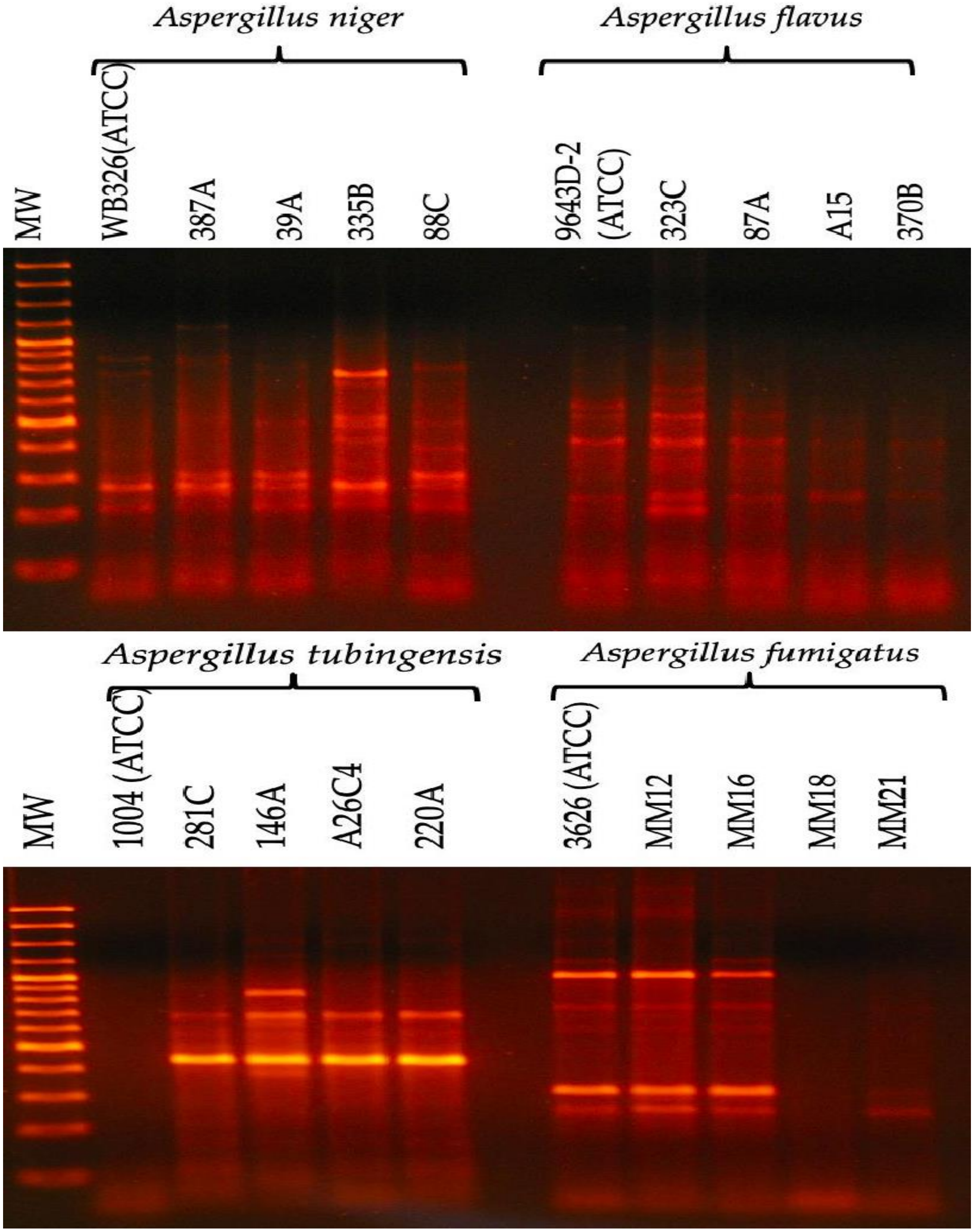


**Figure 39**. Polymorphic patterns obtained by RAPD-PCR for the four species with oligonucleotide B04.

Journal of Fungi

MDPI

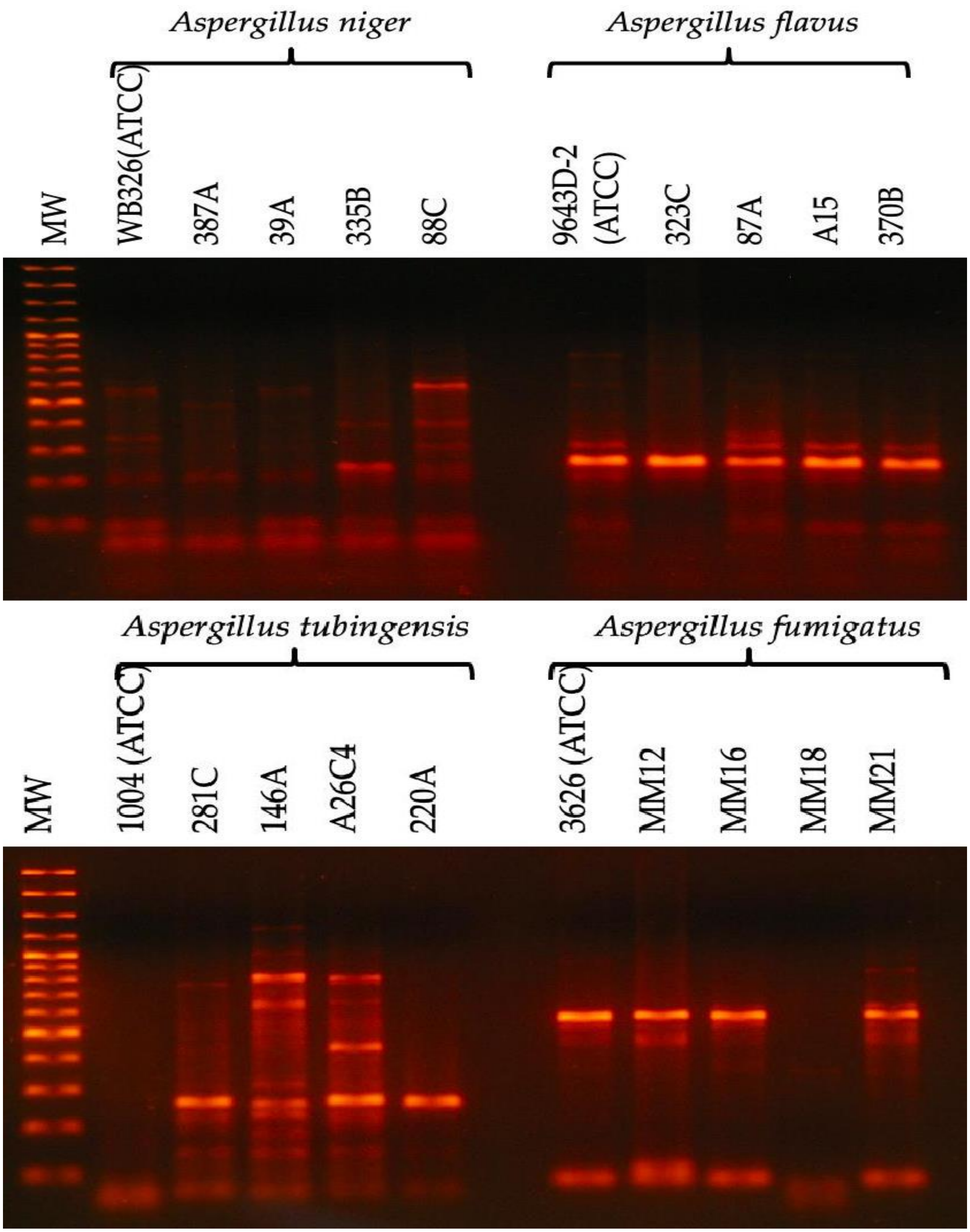


**Figure 40**. Polymorphic patterns obtained by RAPD-PCR for the four species with oligonucleotide OPH-17.

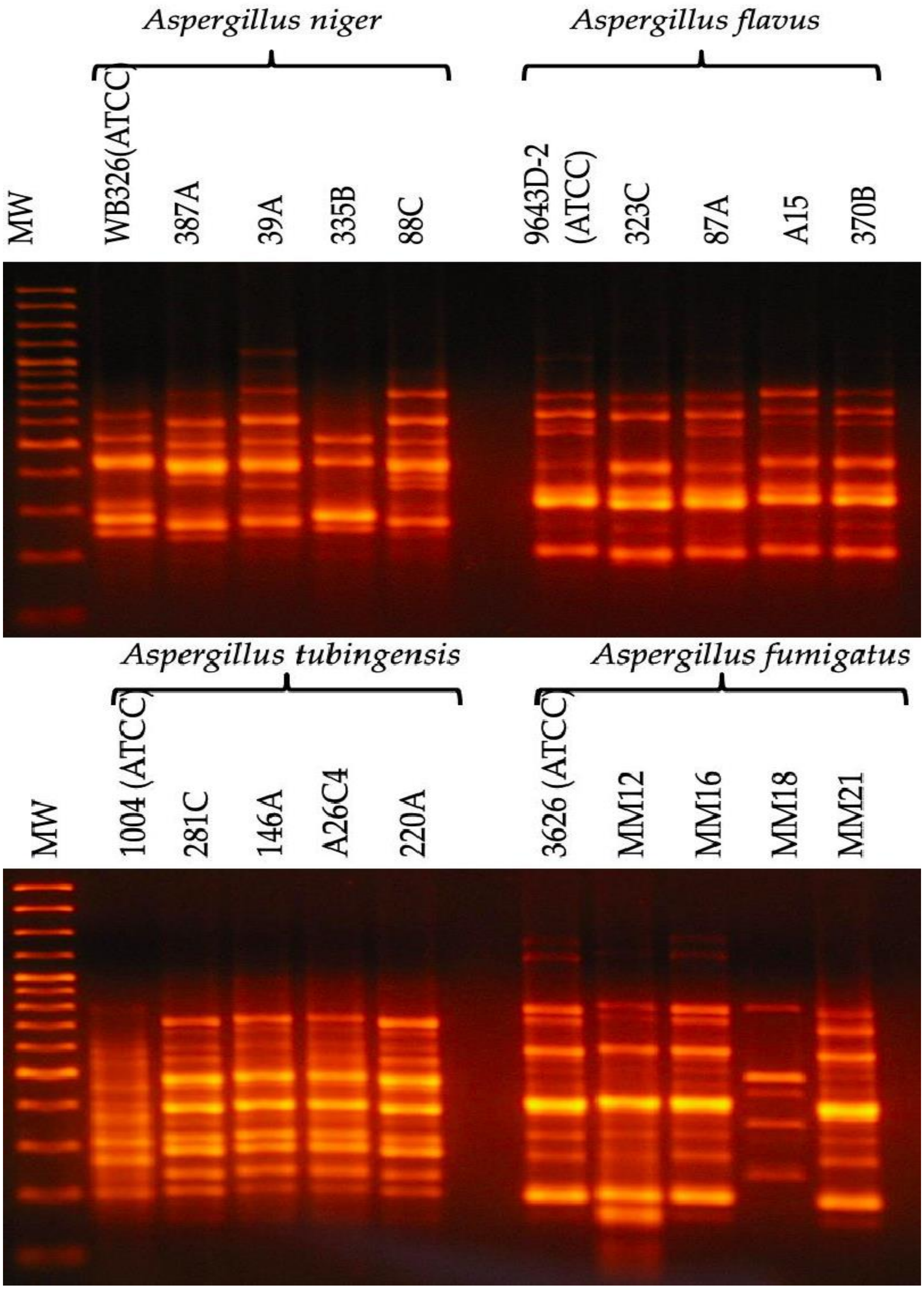


**Figure 41**. Polymorphic patterns obtained by RAPD-PCR for the four species with oligonucleotide T3B.

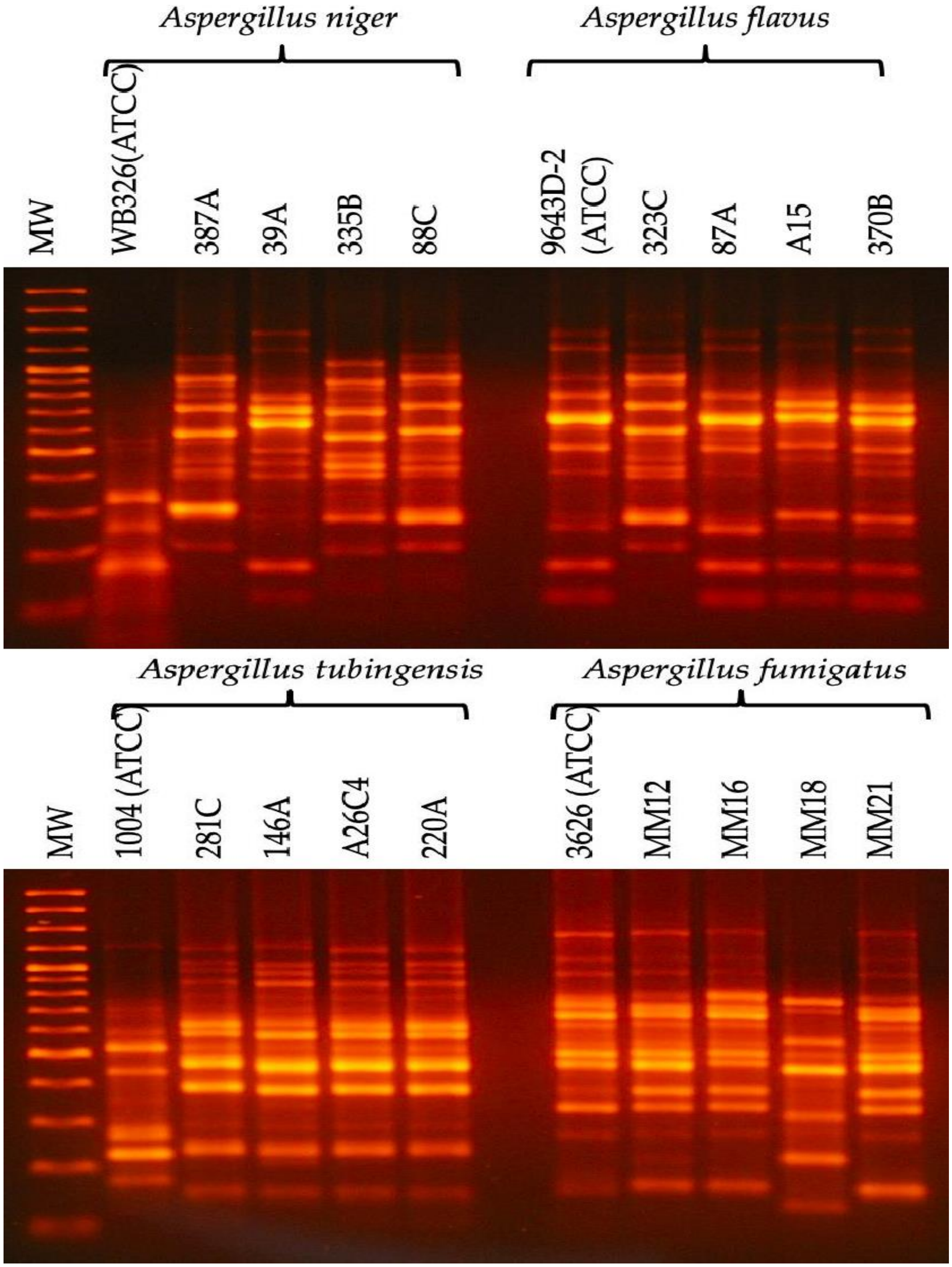


**Figure 42**. Polymorphic patterns obtained by RAPD-PCR for the four species with oligonucleotide OPA-02.

Journal of Fungi

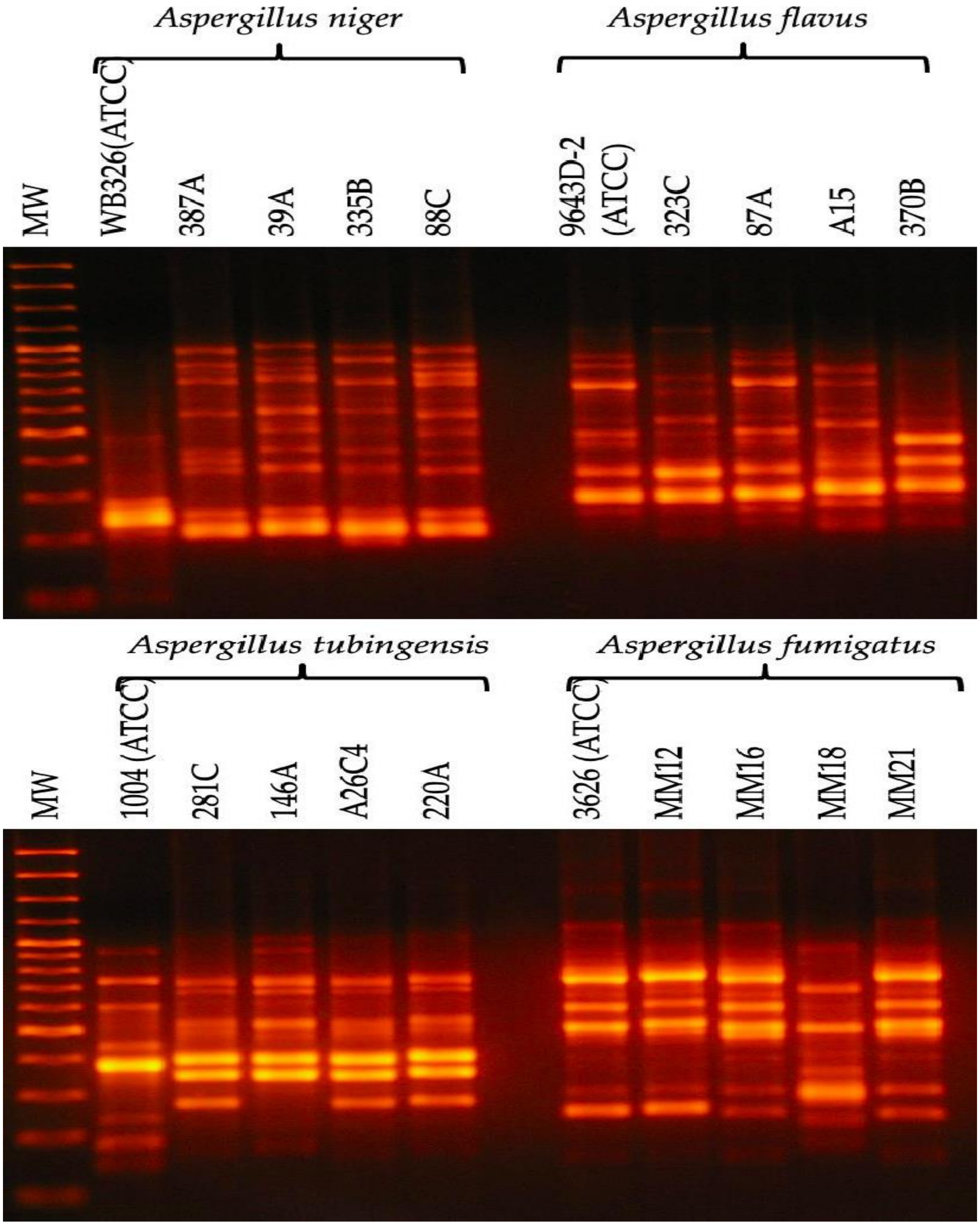


**Figure 43**. Polymorphic patterns obtained by RAPD-PCR for the four species with oligonucleotide OPA-03.
