# Supplementary material for: Selection of Polymorphic Patterns Obtained by RAPD-PCR through Qualitative and Quantitative Analyses to Differentiate Aspergillus fumigatus
Source: J Fungi (Basel). 2022 Mar 13;8(3):296. doi: 10.3390/jof8030296 (PMC8949193; doi:10.3390/jof8030296)
Supplement: Supplementary file 1 [file jof-08-00296-s001.zip › Supplementary Figure S3.pdf]

Journal of Fungi

MDPI

Figures S3 (1-15). Probability equation of the selected primers

OPC-06

$$If\begin{pmatrix} MARCADOR == \text{"OPC-06"} \Rightarrow -21.00226769 + 3.0382244686 * NUMERO\ DE\ BANDAS + -0.47340996 * INTESIDAD + -0.001355211 * pb \\ else \Rightarrow . \end{pmatrix}$$

Figure 1. Linear equation for [*Flavus*] by primer OPC-06.

$$If\begin{pmatrix} MARCADOR == \text{"OPC-06"} \Rightarrow -4.304772735 + 0.5548339918 * NUMERO\ DE\ BANDAS + 0.2661089972 * INTESIDAD + -0.0011350645 * pb \\ else \Rightarrow . \end{pmatrix}$$

Figure 2. Linear equation for [*Fumigatus*] by primer OPC-06.

$$If\begin{pmatrix} MARCADOR == \text{"OPC-06"} \Rightarrow -6.165119829 + 1.1376187607 * NUMERO\ DE\ BANDAS + -0.402930447 * INTESIDAD + -0.000413115 * pb \\ else \Rightarrow . \end{pmatrix}$$

Figure 3. Linear equation for *[Niger*] by primer OPC-06.

$$\text{If}\begin{pmatrix} MARCADOR == \text{"OPC-06"} \Rightarrow \dfrac{1}{\begin{pmatrix} 1 \\ +Exp\,(-Lineal\,[FLAVUS]PorMarcador) \\ +Exp\,(Lineal\,[FUMIGATUS]PorMARCADOR - Lineal[FLAVUS]PorMARCADOR \\ +Exp\,(Lineal[NIGER]PorMARCADOR - Lineal[FLAVUS]PorMARCADOR \end{pmatrix}} \\ else \Rightarrow . \end{pmatrix}$$

Figure 4. Probability (correspondence) that the isolates correspond to [*Flavus*] by primer OPC-06.

$$If\begin{pmatrix} MARCADOR == \text{"OPC-06"} \Rightarrow ifMax\begin{pmatrix} Prob[FLAVUS]PorMARCADOR & \Rightarrow & \text{"FLAVUS"} \\ Prob[FUMIGATUS]PorMARCADOR & \Rightarrow & \text{"FUMIGATUS"} \\ Prob[NIGER]PorMARCADOR & \Rightarrow & \text{"NIGER"} \\ Prob[TUBINGENSIS]PorMARCADOR & \Rightarrow & \text{"TUBINGENSIS"} \\ else & \Rightarrow & \text{""} \end{pmatrix} \\ else \Rightarrow . \end{pmatrix}$$

Figure 5. Equation to determine the most probable species by marker OPC-06.

OPF-01

$$If\begin{pmatrix} MARCADOR == \text{"OPF-01"} \Rightarrow 0.8818445222 + -0.01147971 * NUMERO\ DE\ BANDAS + -0.507586078 * INTESIDAD + -0.000368841 * pb \\ else \Rightarrow . \end{pmatrix}$$

Figure 6. Linear equation for [*Flavus*] by primer OPF-01.

$$If\begin{pmatrix} MARCADOR == \text{"OPF-01"} \Rightarrow -383.3638226 + 44.898815291 * NUMERO\ DE\ BANDAS + 3.5088227063 * INTESIDAD + 0.0003966942 * pb \\ else \Rightarrow . \end{pmatrix}$$

Figure 7. Linear equation for [*Fumigatus*] by primer OPF-01

$$If\begin{pmatrix} MARCADOR == \text{"OPF-01"} \Rightarrow 1.5081763687 + -0.344162387 * NUMERO\ DE\ BANDAS + -0.134566159 * INTESIDAD + 0.0005784623 * pb \\ else \Rightarrow . \end{pmatrix}$$

Figure 8. Linear equation for [*Niger*] by primer OPF-01.

$$If\begin{pmatrix} MARCADOR == \text{"OPF-01"} \Rightarrow \dfrac{1}{\dfrac{1}{\begin{pmatrix} +Exp\,(Lineal\,[FLAVUS]PorMarcador - Lineal[FUMIGATUS]PorMARCADOR) \\ +Exp\,(-\,Lineal\,[FUMIGATUS]PorMARCADOR) \\ +Exp\,(Lineal[NIGER]PorMARCADOR - Lineal[FUMIGATUS]PorMARCADOR) \end{pmatrix}}} \\ else \Rightarrow . \end{pmatrix}$$

Figure 9. Probability (correspondence) that the isolates correspond to [*Fumigatus*] by primer OPF-01.

$$If\begin{pmatrix} MARCADOR == \text{"OPC-06"} \Rightarrow ifMax\begin{pmatrix} Prob[FLAVUS]PorMARCADOR & \Rightarrow & \text{"FLAVUS"} \\ Prob[FUMIGATUS]PorMARCADOR & \Rightarrow & \text{"FUMIGATUS"} \\ Prob\,[NIGER]PorMARCADOR & \Rightarrow & \text{"NIGER"} \\ Prob[TUBINGENSIS]PorMARCADOR & \Rightarrow & \text{"TUBINGENSIS"} \\ else & \Rightarrow & \text{""} \end{pmatrix} \\ else \Rightarrow . \end{pmatrix}$$

Figure 10. Equation to determine the most probable species by marker OPF-01.

OPG-13

$$If\begin{pmatrix} MARCADOR == \text{"OPG-13"} \Rightarrow -4.62316397 + 1.7673994246 * NUMERO\ DE\ BANDAS + -1.214131683 * INTESIDAD + -0.003514185 * pb \\ else \Rightarrow . \end{pmatrix}$$

Figure 11. Linear equation for [*Flavus*] by primer OPG-13.

$$If\begin{pmatrix} MARCADOR == \text{"OPG-13"} \Rightarrow -7.654680371 + 2.2789634365 * NUMERO\ DE\ BANDAS + -0.990792157 * INTESIDAD + -0.0047291 * pb \\ else \Rightarrow . \end{pmatrix}$$

Figure 12. Linear equation for [*Fumigatus*] by primer OPG-13.

$$If\begin{pmatrix} MARCADOR == \text{"OPG-13"} \Rightarrow -10.63447419 + 2.6631351049 * NUMERO\ DE\ BANDAS + -1.234220583 * INTESIDAD + -0.003191852 * pb \\ else \Rightarrow . \end{pmatrix}$$

Figure 13. Linear equation for [*Niger*] by primer OPG-13.

$$\text{If}\begin{pmatrix} MARCADOR == \text{"OPG-13"} \Rightarrow \dfrac{1}{\begin{pmatrix} 1 \\ +Exp\left(Lineal\left[FLAVUS\right]PorMARCADOR\right) \\ +Exp\left(-Lineal\left[FUMIGATUS\right]PorMARCADOR\right) \\ +Exp\left(Lineal\left[NIGER\right]PorMARCADOR\right) \end{pmatrix}} \\ \\ else \Rightarrow . \end{pmatrix}$$

Figure 14. Probability (correspondence) that the isolates correspond to [*Tubingesis*] by primer OPG-13.

$$If\begin{pmatrix} MARCADOR == \text{"OPG-13"} \Rightarrow ifMax\begin{pmatrix} Prob[FLAVUS]PorMARCADOR & \Rightarrow & \text{"FLAVUS"} \\ Prob[FUMIGATUS]PorMARCADOR & \Rightarrow & \text{"FUMIGATUS"} \\ Prob[NIGER]PorMARCADOR & \Rightarrow & \text{"NIGER"} \\ Prob[TUBINGENSIS]PorMARCADOR & \Rightarrow & \text{"TUBINGENSIS"} \\ else & \Rightarrow & \text{""} \end{pmatrix} \\ \\ else \Rightarrow . \end{pmatrix}$$

Figure 15. Equation to determine the most probable species by marker OPG-13.
